# Global and local functions of the Fused kinase ortholog CdaH in intracellular patterning in *Tetrahymena*

**DOI:** 10.1242/jcs.261256

**Published:** 2023-10-04

**Authors:** Chinkyu Lee, Wolfgang Maier, Yu-Yang Jiang, Kentaro Nakano, Karl F. Lechtreck, Jacek Gaertig

**Affiliations:** ^1^ Department of Cellular Biology, University of Georgia, Athens, GA 30602, USA; ^2^ Bioinformatics, University of Freiburg, 79110 Freiburg, Germany; ^3^ Degree Programs in Biology, Graduate School of Science and Technology, University of Tsukuba, Tsukuba, Ibaraki 305-8572, Japan

**Keywords:** Cell cortex, Ciliate, Patterning, Polarity

## Abstract

Ciliates assemble numerous microtubular structures into complex cortical patterns. During ciliate division, the pattern is duplicated by intracellular segmentation that produces a tandem of daughter cells. In *Tetrahymena thermophila*, the induction and positioning of the division boundary involves two mutually antagonistic factors: posterior CdaA (cyclin E) and anterior CdaI (Hippo kinase). Here, we characterized the related *cdaH-1* allele, which confers a pleiotropic patterning phenotype including an absence of the division boundary and an anterior–posterior mispositioning of the new oral apparatus. CdaH is a Fused or Stk36 kinase ortholog that localizes to multiple sites that correlate with the effects of its loss, including the division boundary and the new oral apparatus. CdaH acts downstream of CdaA to induce the division boundary and drives asymmetric cytokinesis at the tip of the posterior daughter. CdaH both maintains the anterior–posterior position of the new oral apparatus and interacts with CdaI to pattern ciliary rows within the oral apparatus. Thus, CdaH acts at multiple scales, from induction and positioning of structures on the cell-wide polarity axis to local organelle-level patterning.

## Teaser

**Summary:** CdaH, a conserved Fused kinase, acts at multiple stages and scales to generate a pattern of cortical organelles during cell division in the ciliate *Tetrahymena thermophila*.

## INTRODUCTION

**Fig. 1.**
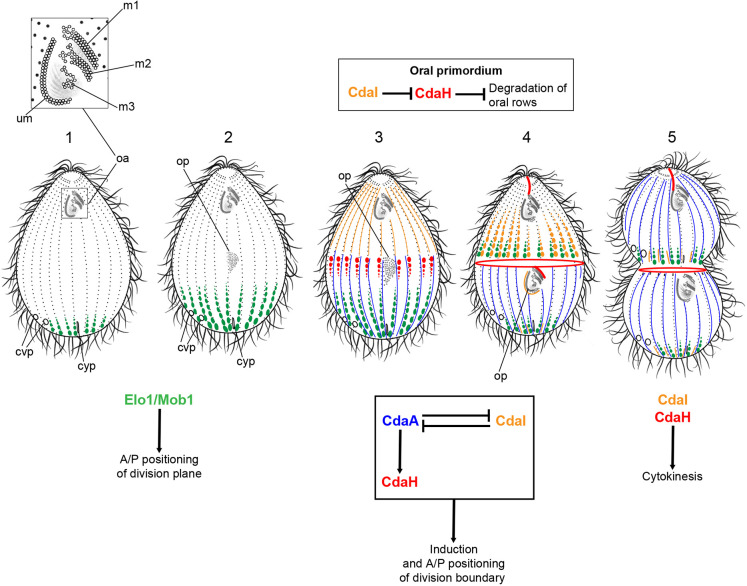
**Anterior-posterior patterning during cell division in *Tetrahymena*.** The diagram (modified from fig. 11 in Cole and Gaertig, 2022 with permission from Wiley; ©2022 The Authors. Journal of Eukaryoti Microbiology published by Wiley Periodicals LLC on behalf of International Society of Protistologists) summarizes the course of cell division in *Tetrahymena*, the localizations of CdaH and other pattern regulators and interactions among them. Stages: 1, interphase; 2, early oral primordium (OP) development; 3, midstage of OP development (shortly before the cortical subdivision); 4, cortical subdivision; and 5, cytokinesis and morphogenesis of new cell ends. A/P, anterior–posterior; cvp, contractile vacuole pores; cyp, cytoproct; m1, m2 and m3, membranelles; oa, oral apparatus; op, oral primordium; um, undulating membrane.

Ciliates have two permanent cell polarity axes: anterior–posterior and circumferential (left–right) (Fig. 1). In *Tetrahymena thermophila*, the oral apparatus (OA) is located near the anterior cell end, whereas the osmoregulatory contractile vacuole pores and the cytoproct (where spent food vacuoles are egested) are located near the posterior cell end, but at different circumferential positions. As proposed by V. Tartar based on his famous microsurgical studies of *Stentor coeruleus*, ciliates divide by a process that amounts to a developmental segmentation of a single cell (Tartar, 1968). The extensive cortical remodeling that takes place throughout the midbody of the dividing ciliate is remarkably precise and, consequently, in the population, the cortical pattern is nearly invariant, which has enabled isolation of unique intracellular patterning mutants (reviewed in Frankel, 1989, 2008). Recently, reverse and forward genetic approaches (Galati et al., 2014) have enabled identification of a number of gene products that contribute to patterning in ciliates (reviewed in Cole and Gaertig, 2022). On the anterior–posterior axis, several highly conserved kinases and kinase regulators control the positions of forming cortical structures, including Elo1 (Lats kinase) (Jiang et al., 2019a), CdaI (Hippo kinase) (Jiang et al., 2017), Mob1 (Slabodnick et al., 2014; Tavares et al., 2012) and CdaA (cyclin E) (Jiang et al., 2020). These proteins appear to form a signaling ‘prepattern’ that acts by localized inhibition: forming structures are excluded from the cortical areas where these prepatterning factors are enriched.

The generation of the division boundary (DB) at the equator of the ciliate parental cell is a key step that precedes cytokinesis and morphogenesis of new cell ends. In *T. thermophila*, loss-of-function mutations of either the posteriorly enriched CdaA or the anteriorly enriched CdaI cause either a failure or a shift in the position of the DB. CdaA and CdaI engage in a mutual antagonism that induces and positions the DB (Jiang et al., 2017, 2020). Here, we investigate a closely related conditional allele, *cdaH-1*, the molecular identity of which has remained unknown since its original description over 40 years ago (Frankel et al., 1980). *cdaH-1* confers a pleiotropic patterning phenotype that includes an absence of the DB and an anterior displacement and partial resorption of the new OA (Frankel, 2008; Frankel et al., 1980). We show that *CDAH* encodes an ortholog of the highly conserved *Drosophila* Fused (Fu) and human Stk36 kinases. Our observations suggest that CdaH contributes to both global (cell-wide) and local (organelle-level) intracellular patterning.

## RESULTS

### The pleiotropic phenotype of *cdaH-1*

In *Tetrahymena*, the anterior–posterior cell polarity is reflected by the different curvatures of cell extremities and asymmetrically placed structures: the anterior OA and the posterior contractile vacuole pores and cytoproct (Fig. 1, stage 1). The main elements of the cortical pattern are the basal bodies (BBs), most of which are ciliated. The majority of BBs are aligned in ∼20 longitudinal rows, whereas a subset of BBs forms four short oral rows. Cell division is initiated with assembly of the new OA (oral primordium, OP) at the subequatorial position on the ventral side (Fig. 1, stage 2). The OP starts as a group of randomly oriented BBs that gradually align into four oral rows: three diagonally oriented membranelles (M1, M2 and M3) and the undulating membrane (UM) (Fig. 1, stages 2 and 3) (Bakowska et al., 1982a). Subsequently, the DB forms at the cell equator and anteriorly to the OP (Fig. 1, stage 4). The DB starts as an array of gaps in all ciliary rows that circumvent the cell (the stage of ‘cortical subdivision’) (Frankel et al., 1981). The sculpturing of new cell ends occurs concurrently with cytokinesis and, eventually, two complete daughters emerge and split apart (Fig. 1, stage 5). The micronucleus divides (by mitosis) at the time of cortical subdivision, whereas the macronucleus divides (by amitosis) concurrently with cytokinesis (Jerka-Dziadosz et al., 2001; Kirk et al., 2008).

**Fig. 2.**
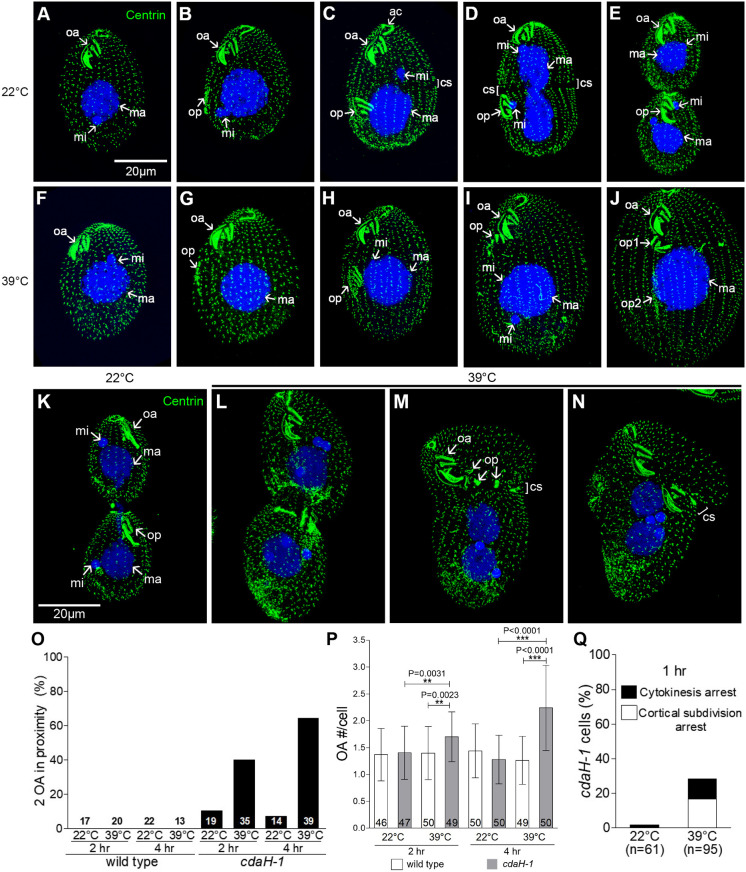
***cdaH-1* confers temperature-sensitive defects in patterning during cell division.** (A–N) Representative SR-SIM images of *cdaH-1* mutants labeled with the 20H5 anti-centrin antibody (green) and DAPI (blue), grown at either 22°C (A–E,K) or 39°C (F–J,L–N). Cells documented in panels A–J,O,P were exposed to 39°C for 3 h, whereas those shown in panels K–N,Q were exposed to 39°C for 1 h. The images are representative of three (A–J) and two (K–N) independent experiments, respectively. ac, apical crown; cs, cortical subdivision; ma, macronucleus; mi, micronucleus; oa, oral apparatus; op, oral primordium; op1, op2, first and second-generation oral primordium, respectively. Scale bars: 20 μm. (O–Q) Quantitative analyses of phenotypic defects in the *cdaH-1* mutants. (O) The frequencies of cells with an OP abnormally close to the old OA. (P) The average number of OAs (old and new) per cell. The asterisks mark significant differences between the datasets (*P*<0.05 using a two-tailed unpaired *t*-test). The bars represent mean±s.d. The numbers of cells scored are displayed. (Q) The graph documents the frequency of *cdaH-1* cells blocked either before or after the stage of cortical subdivision (during cytokinesis). The quantitative data shown in panels O–Q were obtained by scoring dividing cells in two independent experiments.

The overall cortical pattern can be assessed using the 20H5 anti-centrin monoclonal antibody (Salisbury et al., 1988), which marks the BBs. At 22°C, most of the *cdaH-1* homozygotes divided normally (Fig. 2A–E). At 39°C, the *cdaH-1* cells assembled an OP (Fig. 2F,G), but most mutants failed to develop the cortical subdivision (Fig. 2H,I, compare to Fig. 2C,D). The OP displaced anteriorly to land in the vicinity of the old OA (‘op’ in Fig. 2I, also Fig. 2O). In the displaced OP, the oral rows were partially degraded and mispositioned (‘op’ in Fig. 2I). The micronucleus divided but the macronuclear amitosis failed (‘ma’ in Fig. 2I, compare to Fig. 2D). The cell division-blocked *cdaH-1* cells entered the next cell cycle and developed a second-generation OP (‘op2’ in Fig. 2J, also Fig. 2P). In a minority of mutants, the cortical subdivision formed and the macronucleus divided, but there was a subsequent arrest in cytokinesis, followed by translocation of the entire cortices of hemi-cells that produced variable cell morphologies (Fig. 2L–N, compare to Fig. 2K,Q), as already reported for other mutants blocked in cytokinesis (Brown et al., 1999; Williams et al., 2006).

### CdaH is a Fused/Stk36 kinase ortholog

**Fig. 3.**
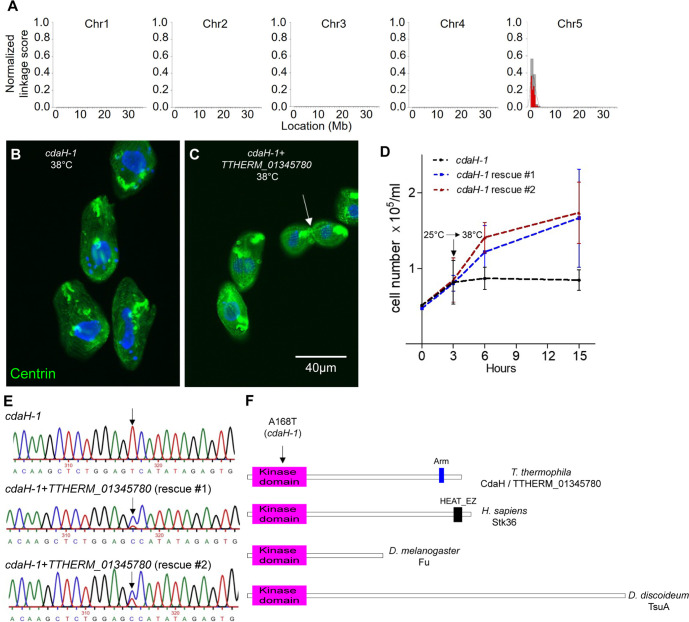
**Mapping of the *cdaH-1* causal mutation to *TTHERM_01345780*.** (A) Mapping of genomic sequence variants linked to *cdaH-1* onto the micronuclear genome by the ACCA method. (B–D) A wild-type *TTHERM_01345780* fragment rescued the *cdaH-1* phenotype by homologous DNA recombination. *cdaH-1* negative control (B) and rescue clone cells (C) maintained at 38°C and fluorescently labeled using anti-centrin (green) and DAPI (blue). Note a dividing cell with a proper fission line in the rescue population (arrow). The images are representative of three experiments. Scale bar: 40 μm. (D) Culture growth curves of *cdaH-1* cells (IA450) and two *cdaH-1+TTHERM_01345780* rescue clones. The cultures were initially grown at 25°C and transferred to 38°C at the time indicted by the arrow. The data points and error bars are the mean±s.d. from three independent experiments. (E) DNA sequencing chromatograms of an amplified region of *TTHERM_01345780* containing the *cdaH-1*-linked variant (arrows). The top panel is the original sequence in the *cdaH-1* mutant (IA450), whereas the two bottom panels show the same sequence in the two independent rescue clones. (F) Domain organization of CdaH and Fused (Fu)/Stk36 orthologs.

*cdaH-1* is a recessive allele on the micronuclear chromosome 5 (Frankel, 2008; Frankel et al., 1980). To map *cdaH-1* by next-generation sequencing, we outcrossed *cdaH-1* homozygotes (strain IA450) and the resulting F1 heterozygotes were crossed to each other to obtain F2 progeny. We pooled a number of phenotypically mutant and wild-type F2s, sequenced the pooled genomes and used the allelic composition contrast analysis (ACCA) bioinformatics workflow (Jiang et al., 2017) to search for genomic variants that co-segregated with the mutant phenotype. A strong ‘variant-to-phenotype’ linkage signal was detected near the end of the left arm of chromosome 5 at ∼0.05 Mb (Fig. 3A). Within this genomic region, two homozygous variants were found that were predicted to affect gene products: G/A at chr5:49333 and C/T at chr5:501775. The latter variant results in a V1176I substitution in TTHERM_ 00649260, a protein with homology to the mammalian centrosomal protein Ccdc81 (Firat-Karalar et al., 2014). The former variant results in a A168T substitution in TTHERM_01345780, a protein kinase. We attempted to rescue the multiplication arrest of *cdaH-1* mutants by biolistic introduction of wild-type fragments spanning the variant position in either *TTHERM_01345780* or *TTHERM_ 00649260*, followed by screening for clones capable of multiplying at the restrictive temperature. Using ∼5×10^6^ mutant cells, two rescue clones were obtained with the *TTHERM_01345780* but not the *TTHERM_ 00649260* fragment (Fig. 3B–D)*.* Sequencing of *TTHERM_01345780* in the two rescue clones revealed both a variant and a wild-type base, consistent with partial replacement of the 90 gene copies in the macronucleus (Zhou et al., 2022) (Fig. 3E). Next, we edited *TTHERM_01345780* in the *cdaH-1* homozygotes by homologous DNA recombination using fragments carrying a portion the 5′ untranslated region (UTR) (with an embedded *neo5* marker) and a portion of the adjacent coding region that terminated either 63 bp upstream or 568 bp downstream of codon 168 (see Materials and Methods for details). At the restrictive temperature of 38°C, all tested paromomycin-resistant transformants that integrated the DNA fragment covering codon 168 proliferated (*n*=95), whereas no rescues were observed in transformants that integrated a fragment that terminated upstream of codon 168 (*n*=96). We conclude that the causal mutation for *cdaH-1* is the A168T substitution in TTHERM_01345780 and the *TTHERM_ 00649260* variant is non-causal but closely linked (in agreement with the estimated rate of meiotic genetic recombination in *T. thermophila*, at ∼50 kb/cM; Orias, 1998).

The *TTHERM_01345780* gene (or *CDAH*, following the gene naming recommendations; Allen et al., 1998) encodes a protein, CdaH, with an N-terminal kinase domain. A Blastp search of the human proteome retrieved Stk36, an ortholog of the ‘segment polarity’ gene product of *Drosophila melanogaster* Fu (Fused, CG6551) (Therond et al., 1993) as the closest match. The amino acid sequence of the kinase domain of CdaH is 64% identical to that of the kinase domain of human Stk36 and 47% identical to that of *D. melanogaster* Fu. In *Dictyostelium discoideum*, TsuA (Tsunami) is a Fused/Stk36 ortholog that is required for cell polarization (Tang et al., 2008) (Fig. 3F). Blastp searches using Fu, Stk36 or TsuA sequences against the predicted *T. thermophila* proteome all returned CdaH as a top match. In a phylogenetic analysis that included the amino acid sequences of 497 human kinase domains (Modi and Dunbrack, 2019b), CdaH formed a statistically supported clade with Stk36 (Fig. S1). Alignment of the entire amino acid sequences of CdaH and established Fused/Stk36 orthologs showed strong homology within and weaker homology outside of the N-terminal kinase domain (Fig. S2). The *cdaH-1* mutation A168T is within the highly conserved ‘APE’ motif located at the end of the kinase activation loop (Modi and Dunbrack, 2019a). Diverse kinases almost invariably have an alanine at this position. Thus, the *cdaH-1* phenotype is likely a result of reduced kinase activity of CdaH. Intriguingly, in mammalian Stk36, the amino acid at the homologous position is a serine. It is possible that Stk36 has weak kinase activity or is a pseudokinase, as is the mammalian paralog Ulk4 (Khamrui et al., 2020). CdaH also has an armadillo repeats domain (Pfam PF00514) near the C-terminus. In Stk36, there is a related C-terminal HEAT domain (Pfam PF02985), which, similar to the armadillo repeats domain, forms a superhelix (Fig. 3F). Overall, CdaH is a compelling ortholog of Fused/Stk36 kinases.

In animals, Fused/Stk36 kinases participate in Hedgehog signaling during embryonic development (reviewed in Maloverjan and Piirsoo, 2012). However, in mammals (Edelbusch et al., 2017; Liu et al., 2016; Nozawa et al., 2013; Wilson et al., 2009) and in the kinetoplastid *Leishmania mexicana* (McCoy et al., 2023), deficiencies in Fused/Stk36 kinases cause structural defects in motile 9+2 cilia, including a failure to assemble central microtubules. *Tetrahymena* assembles numerous motile 9+2 cilia that are used for cell locomotion and feeding. Although the *cdaH-1* mutant phenotype is distinct from the phenotypes reported for ciliary mutants (reviewed in Bayless et al., 2019), we nonetheless examined the *cdaH-1* mutants grown for 3 h at 39°C (a time sufficient to induce cell division arrest) and found no gross defects in the number or length of cilia and in axoneme ultrastructure (Fig. S3).

### During cell division, CdaH accumulates at multiple sites, mostly within the posterior hemi-cell

**Fig. 4.**
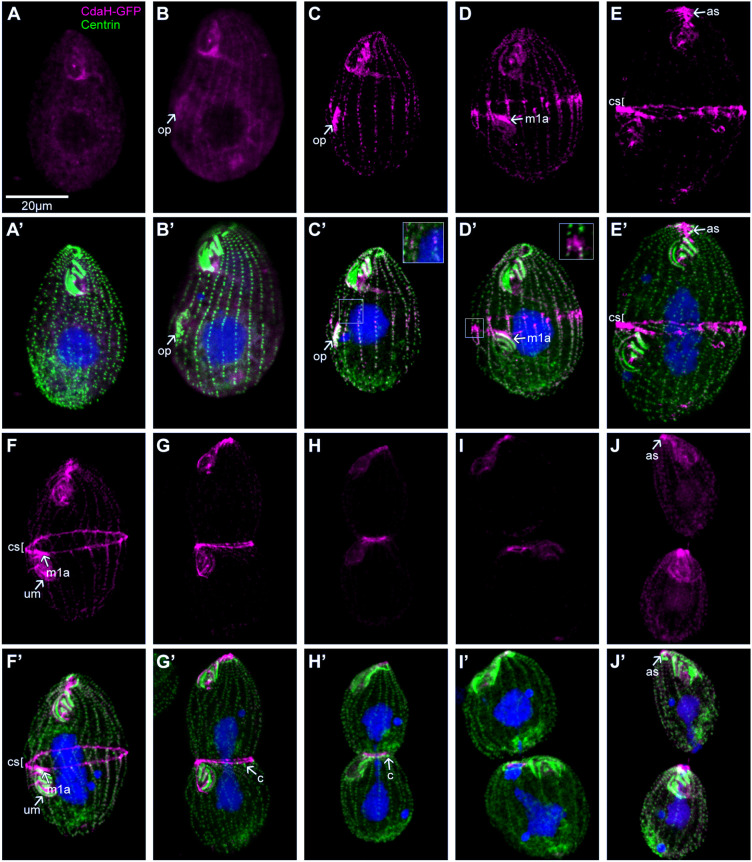
**CdaH–GFP accumulates at multiple locations during cell division.** Confocal images of cells expressing CdaH–GFP, arranged according to the cell cycle stage. The cells were labeled by anti-GFP (magenta) and anti-centrin (green) antibodies and DAPI (blue). (A,A′) Interphase. Note that antibodies frequently bind to the OA, especially its posterior region, resulting in background fluorescence (Jiang et al., 2017). (B–C′) Early OP development. (D,D′) Onset of cortical subdivision. The OP is in an advanced stage, and all rows are visible. (E–H′) Cytokinesis and formation of new cell ends. (I–J′) Scission. The images are representative of five independent immunofluorescence experiments. as, anterior suture; c, BB couplet; cs, cortical subdivision; m1a, M1 arc; op, oral primordium; um, undulating membrane. Scale bar: 20 μm.

**Fig. 5.**
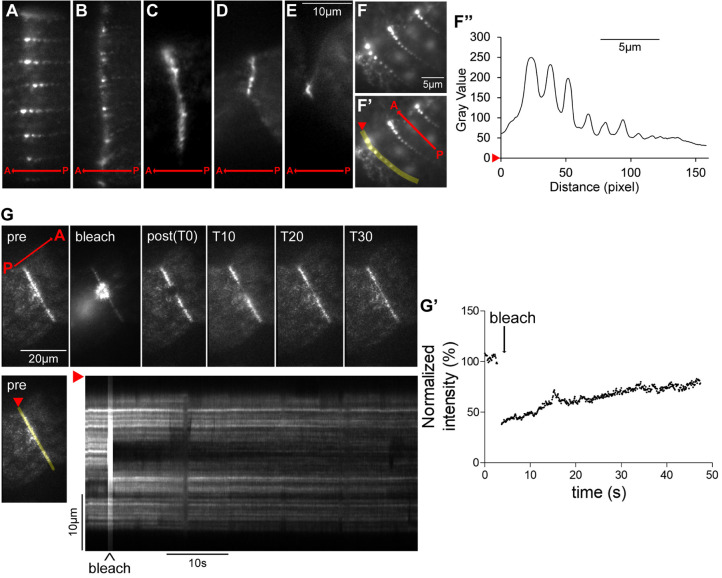
**TIRF imaging of CdaH–GFP.** Live cells expressing CdaH–GFP were imaged after partial immobilization. Red arrows mark the anterior–posterior cell axis. (A–E) Still images of the peri-equatorial region shortly before or at the onset of cortical subdivision (A), and during early (B,C) and late (D,E) stages of cytokinesis. (F–F″) Within the posterior streaks, CdaH–GFP forms a concentration gradient decreasing toward the posterior cell end. F and F′ show the same image. F″ is a signal intensity plot along a single streak marked in F′ with a yellow line. In F′,F″, red arrowheads orient the density plot. (G,G′) Fluorescence recovery after photobleaching (FRAP) experiments reveal that CdaH–GFP within the ring turns over. Following bleaching with a focused laser beam, the signal intensities were analyzed using kymograms. The quantitative analysis of the signal intensity during FRAP is shown in G′. The data are representative of observations done on three cells in the ring stage (in two independent experiments). A, anterior cell end; P, posterior cell end. Scale bars: 10 μm (A–E, kymogram in G); 5 μm (F–F″); 20 μm (G, still images).

We tagged CdaH with GFP at the C-terminus by editing the *CDAH* locus. No CdaH–GFP signal above the background was present in non-dividing cells (Fig. 4A,A′). In early dividers (in which the OP had just started to form), a faint CdaH–GFP signal appeared along the entire length of somatic ciliary rows and in the OP (Fig. 4B,B′, compare to Fig. 4A,A′). Later in development, CdaH–GFP presented as streaks of dots near the BBs of somatic rows and the dot intensity was higher within the posterior hemi-cell; the anterior margin of strong CdaH–GFP signal corresponded to the future plane of cortical subdivision (Fig. 4C,C′; Fig. S4B–B″). At the onset of cortical subdivision, the CdaH–GFP streaks became polarized in signal intensity (Fig. 4D,D′). Live observations using total internal reflection fluorescence (TIRF) microscopy revealed that each streak had multiple dots, the signal intensities of which formed a gradient decreasing toward the posterior cell end (Fig. 5A,F–F″; Movie 1). It has been suggested that in ciliates, the anterior–posterior patterning factors are distributed by motors along the cortical longitudinal microtubules (Cole and Gaertig, 2022; Marshall, 2021). However, kymograms did not reveal signals moving between the dots either within the streaks or between the streaks (Fig. S5A–B′). In contrast, after photobleaching, the dot signals recovered to near pre-bleach level within ∼1 min (Fig. S5C,C′), indicating a rapid turnover, likely by exchange with soluble CdaH in the cell body and possibly with involvement of intracytoplasmic microtubules that connect to the BBs (Gaertig et al., 1993). When the cortical subdivision (‘cs’ in Fig. 4E–F′) was fully developed and cytokinesis and macronuclear amitosis had started, CdaH formed a ring around the cell equator (Fig. 4E–F′). An apparent transition from the streak pattern to the ring pattern was seen by TIRF microscopy (Fig. 5B; Fig. S5B,B′). Super-resolution structured illumination microscopy (SR-SIM) revealed that the CdaH–GFP ring was subequatorial and positioned at the posterior edge of the cortical subdivision (Fig. S4C–D″). At the stage when the ring was fully formed, the most anterior BBs of a subset of the posterior half-rows exist as pairs called BB couplets (marked ‘c’ in Fig. 4G′,H′) (Jerka-Dziadosz, 1981; McCoy, 1974; Numata et al., 1995). Later on, the BB couplets associate laterally to form the new ‘apical crown’ (AC), an incomplete circle of paired BBs that represent the apex of the cell (marked ‘ac’ in Fig. 2C). The CdaH–GFP ring was located immediately anterior to the BB couplets (Fig. 4G′,H′). After photobleaching of a portion of the ring in live cells, the signal recovered to 90% of the pre-bleach level within 50 s, indicating a rapid turnover (Fig. 5G,G′; Movie 2). During the course of cytokinesis, the CdaH–GFP ring narrowed along with the degree of cell constriction (Fig. 4G–H′, Fig. 5C,D; Fig. S4D–E″). When the two daughters were about to split apart, a residual CdaH–GFP ring was present at the anterior tip of the posterior daughter (Fig. 4I–J′, Fig. 5E; Fig. S4E–E″). A distinct CdaH–GFP signal was also present near the old anterior cell end, as a short ribbon near the ends of somatic rows on the ventral right side, partially overlapping with the AC and connecting to the old OA. We will refer to this CdaH–GFP pool as the anterior suture (AS) (‘as’ in Fig. 4E,E′,J,J′; Fig. S4C′,C″,E′,E″). The AS appeared at the same time as the CdaH ring (Fig. 4E,E′, compare to Fig. 4D,D′). These two CdaH–GFP pools (the AS and the ring) could be related, as both were positioned near the anterior ends of ciliary rows (old and new, respectively).

CdaH also localized to the OP and less consistently to the old OA. CdaH–GFP appeared as a uniform signal across the entire OP during the early phase of oral morphogenesis (Fig. 4C,C′). When the rows emerged within the OP, a narrow patch of CdaH–GFP formed anteriorly to the new M1 (most anterior) membranelle. We will refer to this pool of CdaH–GFP as the ‘M1 arc’ (M1A) (labeled ‘m1a’ in Fig. 4D,D′,F,F′; Fig. S4C′,D′,D″). During early cytokinesis, CdaH–GFP also marked the UM (Fig. 4F–G′; Fig. S4D′,D″). Whereas the CdaH population associated with the division plane (streaks and ring) correlates with roles in the cortical subdivision and cytokinesis, the OP-associated CdaH could be involved in OP positioning and morphogenesis (see below).

### CdaH colocalizes with the cytokinetic actin ring at the anterior end of the posterior hemi-cell

**Fig. 6.**
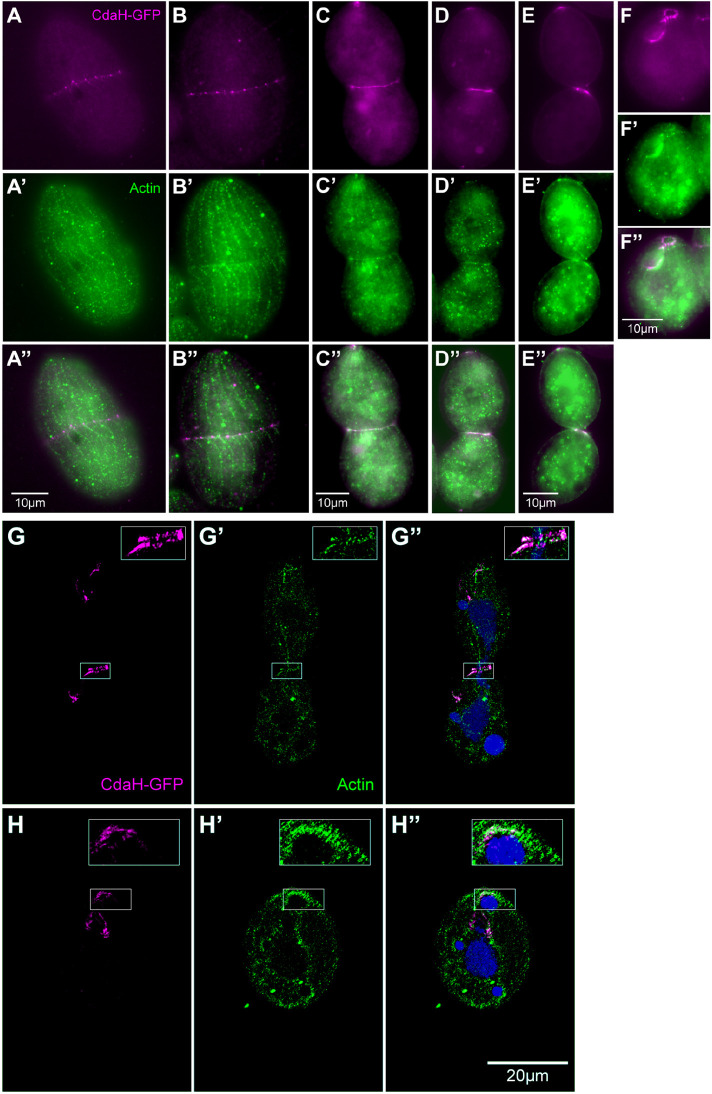
**CdaH–GFP colocalizes with actin at the anterior margin of the posterior daughter cell.** (A–F″) The CdaH–GFP-expressing cells were fixed with cold methanol and labeled with polyclonal anti-Act1 actin antibodies (green) and CdaH–GFP was imaged directly (magenta). (A–A″) Cortical subdivision prior to cytokinesis. (B–E″) Stages of cytokinesis. (F–F″) An early posterior post-divider. The images shown in panels A–F″ are representative of four independent experiments. (G–H″) SR-SIM images of cells fixed with paraformaldehyde in a late stage of cytokinesis around at the time of scission (G–G″) and of a posterior post-divider shortly after scission (H–H″) labeled with anti-GFP (magenta), anti-Act1 (green) and DAPI (blue). Note that, using this fixation method, the direct GFP signal of CdaH–GFP was not visible in the green channel. The images shown in panels G–H″ are representative of two independent experiments. Scale bars: 10 μm (A–F″); 20 μm (G–H″).

The timing and position of the subequatorial CdaH–GFP ring coincides with the assembly of the microfilament-rich cytokinetic ring (Jerka-Dziadosz, 1981; Yasuda et al., 1980). We therefore explored the relationship between the two rings by co-imaging CdaH–GFP and actin (Act1). The CdaH–GFP ring started to form shortly before the actin ring appeared at the onset of cytokinesis (Fig. 6A–B″) and the two rings strictly colocalized during the course of cell constriction based on fluorescence (Fig. 6A–D″) and super-resolution (Fig. 6G–G″) imaging. Strikingly, before the scission of daughter cells, both rings co-localized at the anterior tip of the posterior daughter (Fig. 6E–E″). Moreover, shortly after cytokinesis, residual actin and CdaH–GFP ring signals were present at the tip of the posterior post-divider (Fig. 6F–F″,H–H″). These data indicate that the CdaH ring is associated with or is even a part of the cytokinetic ring. Additionally, in *Tetrahymena*, the contractile ring appears to form and operate at the tip of the posterior hemi-cell throughout cytokinesis.

### Distribution of CdaH within the posterior hemi-cell requires CdaA

**Fig. 7.**
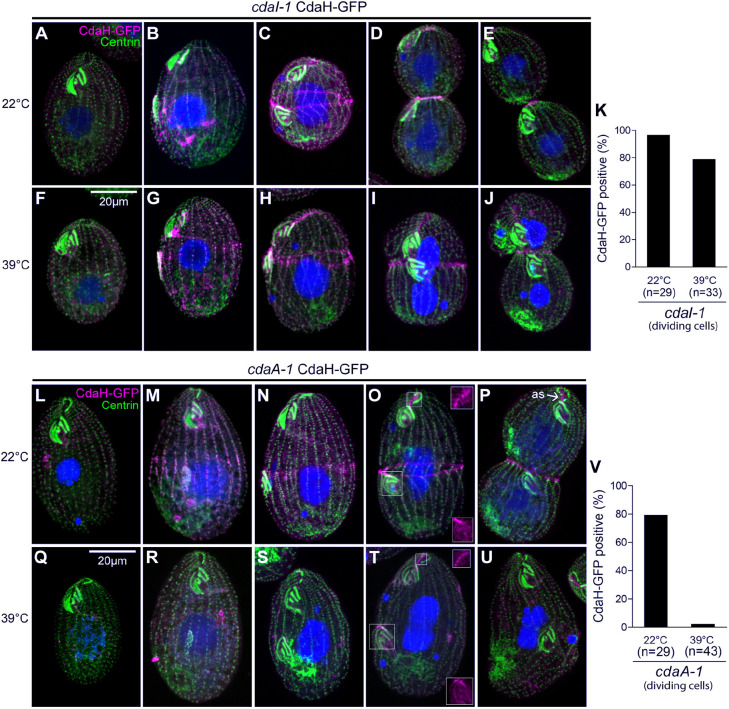
**CdaA, but not CdaI, is required for proper distribution of CdaH.** (A–J) Confocal images of *cdaI-1* cells expressing CdaH–GFP, labeled with anti-GFP (magenta), anti-centrin (green) and DAPI (blue). Prior to staining, cells were incubated at either 22°C (A–E) or 39°C (F–J) for 2 h. (K) The graph shows the percentage of dividing cells with a (stage-appropriate) pattern of CdaH–GFP. (L–U) Confocal images of *cdaA-1* cells expressing CdaH–GFP, labeled with anti-GFP (magenta), anti-centrin (green) and DAPI (blue), incubated at either 22°C (L–P) or 39°C (Q–U) for 2 h. (V) The graph shows the percentage of dividing cells with CdaH–GFP in the form of either posterior streaks or a subequatorial ring (sub-pools associated with the cortical subdivision and cytokinesis). The images shown are representative of three independent experiments. Scale bars: 20 μm.

The anterior CdaI and posterior CdaA are two mutually antagonistic factors that interact to induce and position the DB. One or both of these proteins might interact with CdaH, based on the overlaps in the loss-of-function phenotypes (Fig. S6) and localizations. We therefore examined how the distribution of CdaH–GFP is affected by expression of the loss-of-function alleles *cdaI-1* or *cdaA-1*. In *cdaI-1* homozygotes, at the permissive temperature of 22°C, as expected, the localization pattern of CdaH–GFP was unaffected (Fig. 7A–E,K). At 39°C, the distribution of CdaH–GFP appeared normal except that all pools within the posterior hemi-cell were shifted anteriorly, consistent with the global displacement of the division plane conferred by *cdaI-1* (Fig. 7F–J). It therefore appears that the distribution of CdaH is not directly dependent on CdaI.

In the *cdaA-1* background, at the permissive temperature of 22°C, pattern of CdaH–GFP was unaffected as expected (Fig. 7L–P, see also 7V). At the restrictive temperature of 39°C, the posterior CdaH–GFP signal was greatly diminished (Fig. 7Q–U, compare to Fig. 7L–P, see also 7V). Specifically, both the posterior streaks and the subequatorial ring were not detectable at stages at which they were present at the permissive temperature (Fig. 7R–U, compare to Fig. 7M–P). Interestingly, the AS and OP (including M1A) signals of CdaH–GFP appeared unaffected (insets in Fig. 7T). Thus, the distribution of CdaH–GFP is dependent on CdaA in a subset of locations that are associated with the cortical subdivision and cytokinesis. In a reciprocal experiment, the distribution of CdaA–GFP was unaffected by conditional expression of *cdaH-1* (Fig. S7). These data support the placement of CdaH downstream of CdaA in the context of the formation of the posterior streaks and the subequatorial ring, which correlates with its functions in DB formation and cytokinesis.

### CdaH interacts with CdaI to pattern the oral primordium

**Fig. 8.**
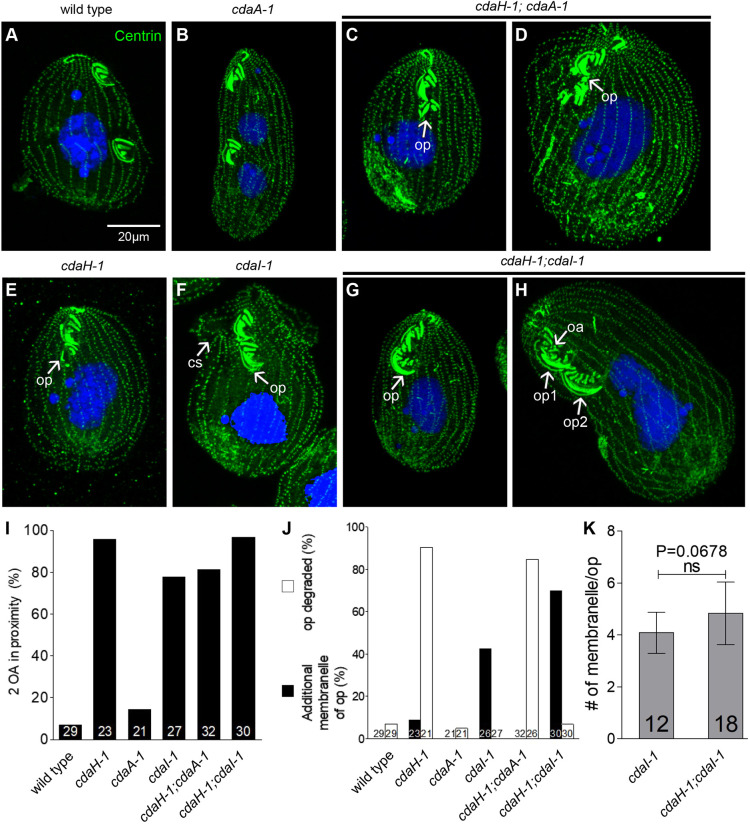
**CdaH interacts with CdaI to shape the developing oral primordium.** (A–H) Representative confocal images of single- and double-mutant cells with the indicated genotypes, labeled with the anti-centrin antibody (green) and DAPI (blue) after incubation for 3–6 h at 39°C. (I–K) The graphs quantify the following phenotypes in the dividing cells at 39°C: the frequency of cells with a displaced OP (I), the frequency of cells with an OP that was either partially degraded or had extra membranelle rows in the OP (J), and the number of membranelles per OP (K). The bars indicate mean±s.d. The numbers of cells scored are displayed on bars. Phenotype scoring was done on dividing cells in two independent experiments. ns, not significant (two-tailed unpaired *t*-test).

To further explore interactions between CdaH and either CdaA or CdaI, we examined the phenotypes of double mutants using loss-of-function alleles: *cdaH-1*, c*daA-1* and *cdaI-1*. Conveniently, all alleles are highly penetrant at 39°C. The phenotype of most of the *cdaH-1;cdaA-1* double homozygotes at 39°C (3–6 h) was similar to that of *cdaH-1* alone: a frequent failure of the DB formation, an anterior OP displacement and partial degradation of the OP (Fig. 8A–E,I,J). Although the double-mutant phenotype did not reveal an interaction, there was no contradiction to the model that CdaH acts downstream of CdaA in the context of the DB formation and cytokinesis, and that CdaH has a CdaA-independent role in OP positioning and stability.

Similarly, the phenotype of the *cdaH-1;cdaI-1* at 39°C resembled that of *cdaH-1* alone, with the cortical subdivision failing to develop in most cells and an extreme anterior displacement of the OP (Fig. 8F–I). Unexpectedly, the OP degradation conferred by *cdaH-1* alone was partially suppressed in the double *cdaH-1;cdaI-1* mutants. In particular, whereas the UM was usually severely degraded in the *cdaH-1* mutants, it remained intact in the double mutants, both in the first- and second-generation OPs (Fig. 8G,H, compare to Fig. 8E; also Fig. 8J). Thus, CdaI activity might inhibit the CdaH activity that prevents OP degradation. Furthermore, the second-generation OP of the *cdaH-1;cdaI-1* double mutants often had an excessive number of membranelles. As an example, in the double-mutant cell shown in Fig. 8H, the second-generation OP (‘op2’) had seven (instead of the normal three) membranelles. Importantly, the allelic *cdaI-2* mutants were reported to have an increased number of membranelles in the OA (Frankel, 2008). Indeed, the *cdaI-1* single mutants used here also had extra membranelles (Fig. 8F,J). Although the addition of *cdaH-1* did not increase the average number of membranelles per OA, it increased the penetrance of the extra-membranelle phenotype (Fig. 8J,K). All these observations indicate that CdaH not only participates in the global positioning of the OP on the anterior–posterior axis, but also contributes to the local patterning of the OP where it might interact with CdaI.

## DISCUSSION

The cortical patterns of ciliates exceed in complexity the most sophisticated intracellular patterns seen in other eukaryotic cells (Frankel, 1989, 2008). Powerful self-organizing mechanisms are likely involved, based on the ability of some ciliates to generate the pattern *de novo* during encystation (Fryd-Versavel et al., 2010; Grimes, 1973a,b) or during regeneration from small cell fragments (Gruber, 1885; Morgan, 1901; Tartar, 1960, 1961). Surprisingly, despite the peculiar features of the ciliate phylum, recent studies in *Tetrahymena* revealed that the positions of forming cortical structures on the anterior–posterior axis are controlled by several conserved kinases and kinase-binding proteins, including orthologs of Hippo signaling components (reviewed in Cole and Gaertig, 2022; Soares et al., 2019). These proteins appear to form a prepattern that excludes forming structures from specific cortical regions. The prepatterning activities occur in two phases. Before the stage of cortical subdivision, the posteriorly enriched ‘early Hippo circuit’ (with Elo1) positions the entire division plane including the OP and DB (Jiang et al., 2019a). Subsequently, a mutually antagonistic pair of the anterior ‘late Hippo circuit’ (with CdaI) and the posterior CdaA induce and position the DB (Jiang et al., 2017, 2020; Tavares et al., 2012). In addition, at the time of the DB appearance, CdaI maintains the subequatorial OP position by preventing its anterior displacement (Jiang et al., 2017). Based on the phenotype of the single available allele (*cdaH-1*), CdaH acts during the late stage that involves CdaI and CdaA. CdaH has multiple functions that can be seen as either global or local. The global function of CdaH is in maintaining the position of the OP in reference to the anterior–posterior cell polarity axis. The local functions involve execution of cortical subdivision and cytokinesis and shaping the OP. CdaH presents at multiple locations that correlate with its multiple patterning functions, offering an explanation for the highly pleiotropic phenotype of *cdaH-1*. These observations agree with the relatively long period of temperature sensitivity for *cdaH-1* expression during cell division compared to that of *cdaA-1* that specifically affects DB formation (Frankel et al., 1980).

### The global function of CdaH: positioning the oral primordium on the anterior–posterior axis

*cdaH-1* confers a dramatic anterior displacement of the OP that phenocopies *cdaI-1* (Frankel, 2008; Jiang et al., 2017). As documented earlier for *cdaI-1*, in the *cdaH-1* mutants, the initial position of the OP is correct and the structure shifts anteriorly later at the time of the DB formation. Both CdaI–GFP and CdaH–GFP are enriched at the OP, whereas CdaA–GFP is not. Thus, there is a correlation between the role of CdaH in OP positioning and presence within the structure. CdaH and CdaI activities might anchor the OP at the correct anterior–posterior position in response to global positioning cues. It remains to be explored whether CdaH and CdaI interact to execute the OP positioning as they do to pattern the internal organization of the OA (see below).

### The local functions of CdaH

#### Shaping the oral primordium

The morphogenesis of the OP is a remarkably complex process of apparent self-organization that includes proliferation of BBs, formation of BB pairs, alignment of BB pairs into rows, emergence of BB triplets and quartets, and resorption of some and movements of other BBs (Bakowska et al., 1982b). The exact course of these events is row specific and, consequently, each row acquires a unique organization. Both CdaI and CdaH localize to the OP but, within the organelle, they occupy non-identical domains. The M1A pool of CdaH–GFP is of particular interest as, to our knowledge, the first row-specific marker. The *cdaI* alleles increase the number of membranelle rows in the OP (Frankel, 2008; Jiang et al., 2017) and we show here that *cdaH-1* increases the penetrance of this phenotype. In contrast, *cdaH-1* confers broad degeneration of oral rows and this phenotype is suppressed by *cdaI-1*. During the normal course of late OP development, a number of BBs at multiple positions (including the right margins of membranelles and the most posterior region of the OP) are resorbed (Bakowska et al., 1982b). The programmed BB resorption could be a part of the mechanism that determines the row number and generates the unique organization of specific oral rows. CdaI and CdaH might act within the OA to control the resorption of BBs.

#### In division boundary formation and cytokinesis

During the course of cell division, the earliest defect conferred by *cdaH-1* is the block in DB formation. We showed previously that the induction and positioning of the DB on the anterior–posterior axis are coupled and involve mutually antagonistic posterior CdaA and anterior CdaI (Jiang et al., 2020). Prior to DB formation, CdaH colocalizes with CdaA to the posterior half-rows in a CdaA-dependent manner. Shortly before the cortical subdivision, CdaH is enriched at several BBs proximal to the cortical gap as a gradient decreasing toward the posterior cell end. The same area contains a posteriorly decreasing gradient of phosphoserine– or phosphothreonine–proline epitopes (Kaczanowska et al., 1999). Thus, CdaH might contribute to generation of the gradient of protein phosphorylation within the subequatorial region that might drive the formation of the DB.

We show that following DB formation, CdaH is required for the execution of cytokinesis and that CdaH forms a ring that colocalizes with the actin contractile ring. As in other eukaryotes, in *Tetrahymena*, the contractile ring contains microfilaments, actin and actin-binding proteins (Edamatsu et al., 1992; Gonda and Numata, 2002; Jerka-Dziadosz, 1981; Numata and Gonda, 2001; Numata et al., 2000; Shirayama and Numata, 2003; Watanabe et al., 1998). Although it is assumed that the microfilaments and myosin motors generate the force for cell constriction, this has not yet been firmly established as ciliates lack myosin-2, the principle force producer in animal cytokinesis (Sugita et al., 2011) and, in *Tetrahymena*, cytokinesis is not sensitive to the actin inhibitor latrunculin A (Shimizu et al., 2013). Nevertheless, cytokinesis is blocked by depletion of cleavage furrow-associated actin-4 in *Paramecium* (Sehring et al., 2007, 2010) and by overexpression of GFP–Act1 in *Tetrahymena* (Hosein et al., 2003). The CdaH ring appears to form shortly before the actin ring, indicating that CdaH promotes contractile ring assembly. Importantly, we found that the contractile ring assembled at the anterior margin of the posterior hemi-cell and its apparent remnant was inherited by the posterior daughter. Thus, in *Tetrahymena* and likely in other ciliates as well, the contractile ring is placed asymmetrically. Interestingly, the anterior tip of an interphase *Tetrahymena* cell contains a band of microfilaments (apical band) that is attached to the most anterior BBs (the AC) (Jerka-Dziadosz et al., 1981, 2001). The two microfilament-rich organelles that form at the new anterior cell end (contractile ring and apical band), might be separate structures. Alternatively, the remnant of the contractile ring might be remodeled into the apical band. Ultrastructural studies are needed to resolve the relationships between CdaH, the contractile ring and the apical band.

### Evolution of Fused kinases

The multifunctionality of CdaH is surprising because the genomes of ciliates encode an extraordinarily large number of kinases (1069 in *T. thermophila*) that arose by gene duplications and likely gained lineage-specific functions (Aury et al., 2006; Eisen et al., 2006; Reiff and Marshall, 2017 preprint; Swart et al., 2013). However, there already are well-documented cases of ‘pleiotropic’ kinases in animals, including the Aurora B and Polo kinases, that play multiple roles during cell division (Hadders and Lens, 2022; Zitouni et al., 2014).

Given that ciliates are an ‘early-branching’ non-opisthokont lineage of eukaryotes (Burki et al., 2020), our studies shed light on the evolution of the Fused kinases. Fused was discovered as a product of a ‘segment polarity’ gene in *Drosophila* (Busson et al., 1988; Gergen and Wieschaus, 1986; Nusslein-Volhard and Wieschaus, 1980; Perrimon and Mahowald, 1987) and, in animals, Fused kinases function in Hedgehog signaling (reviewed in Maloverjan and Piirsoo, 2012). Ciliate genomes appear to lack homologs of proteins that participate in Hedgehog signaling other than Fused. Although Fused is required for proper Hedgehog signaling during embryogenesis in *Drosophila* (Maloverjan and Piirsoo, 2012), in mammals, the Fused ortholog Stk36 plays a non-essential function in Hedgehog signaling (Maloverjan and Piirsoo, 2012), possibly redundant with Ulk3 (Han et al., 2019). Intriguingly, in mice, Stk36 and a closely related kinase, Ulk4, are required for assembly of central microtubules in the 9+2 cilia (Liu et al., 2016; Nozawa et al., 2013; Wilson et al., 2009). The ciliary role of Fused kinases could be ancestral, based on the recent report that Fused and Ulk4 are required for correct assembly of flagellar axonemes in the kinetoplastid *Leishmania* (McCoy et al., 2023). Although our study did not reveal a ciliary function for CdaH, the genome of *Tetrahymena* encodes orthologs of Ulk4 (TTHERM_00756520 and TTHERM_00726480) that could fulfill the ciliary roles (Fig. S1). If such a division of labor exists, the *Tetrahymena* model could be useful in dissecting the ciliary versus non-ciliary functions of Fused-related kinases.

We reveal here a role for Fused in intracellular patterning. In *Dictyostelium*, the Fused ortholog TsuA is required for cell polarization during developmental aggregation, and, intriguingly, TsuA colocalizes with microtubules (Tang et al., 2008). In *Drosophila,* Fused phosphorylates Cos2 kinesin to activate Hedgehog signaling (Nybakken et al., 2002; Raisin et al., 2010; Ruel et al., 2007). In *Arabidopsis thaliana*, the Fused ortholog TIO is required for expansion of the microtubule-rich phragmoplast during cytokinesis and associates with two members of the kinesin-12 family (Oh et al., 2012). CdaH is closely associated with the microtubule-rich BBs. As suggested by others (Oh et al., 2012; Tang et al., 2008), the evolutionarily conserved molecular function of Fused kinases could be to phosphorylate microtubule motors and, specifically, kinesins. In addition, studies in *Dictyostelium* and our work implicate Fused kinases in cell polarity-related phenomena: formation of the cell polarity axis (Tang et al., 2008) and positioning and patterning of organelles (this study). It is possible that Fused orthologs have conserved roles in distributing polarity-related molecules along microtubules via regulation of kinesin motors. Such activities could also be behind the role of Fused homologs in the assembly of ciliary axonemes (Liu et al., 2016; McCoy et al., 2023; Nozawa et al., 2013; Wilson et al., 2009). The genome of *Tetrahymena* encodes an unusually large number of kinesins (77) (Wickstead and Gull, 2006) and, therefore, some could participate in intracellular positioning as phosphorylation targets of CdaH. However, our study also points to a close relationship of Fused with actin.

## MATERIALS AND METHODS

### *Tetrahymena* strains

All strains were obtained from the *Tetrahymena* Stock Center (Cornell University, Ithaka, NY, USA). CU428 (TSC_SD00178) was used as the wild type. IA450 (TSC_SD01593) is homozygous for *cdaH-1*, IA104 (TSC_SD01447) is homozygous for *cdaA-1*, and IA237 (TSC_SD01449) is homozygous for *cdaI-1* (Frankel, 2008; Jiang et al., 2017). Double-mutant homozygotes were generated by crosses to create double heterozygotes, and self-crosses using B*VII (TSC_SD00023) to generate micronuclear homozygotes. The micronuclear genotypes of the self-cross progeny were determined using outcrosses to single-mutant homozygotes. Pairs of strains with the desired micronuclear genotypes were crossed to each other to obtain double-mutant homozygote progeny. The genotypes of final double homozygotes were verified by PCR amplification and Sanger DNA sequencing across the base positions of mutant alleles. Strains were cultured in SPP medium (1% proteose–peptone, 0.2% dextrose, 0.1% yeast extract and 0.003% EDTA:ferric sodium salt) supplemented with antibiotics (SPPA medium) (Gaertig et al., 2013; Gorovsky, 1973).

### Mapping of *cdaH-1* by comparative next-generation sequencing

We applied the ACCA workflow (Jiang et al., 2017) to map the causal mutation for *cdaH-1*. IA450 was crossed to CU427 [strain homozygous for *chx1-1* a cycloheximide (cy)-resistant allele in the micronucleus, TSC_SD00715], and the resulting F1s presumed to be double heterozygotes (*cdaH-1*/*CDAH*; *chx1-1*/*CHX1*) were allowed to undergo phenotypical assortment to cy sensitivity by multiplication for ∼60 generations in a non-selective medium. Subsequently we crossed several F1s in multiple combinations and selected F2 progeny using cy resistance (15 µg/ml). We picked 38 and 31 F2s with a *cdaH-1* and wild-type phenotype, respectively, and pooled them according to the phenotype. The mutant and wild-type F2 pools were cultured to a mid-log phase in 25 ml of SPPA medium at room temperature, starved for 2 days also at room temperature in 60 mM Tris-HCl, pH 7.5, and total genomic DNA was extracted from each pool using the urea method (Gaertig et al., 1994). Genomic libraries were constructed with the Illumina Truseq primer adapters and sequenced on an Illumina HiSeq X, which generated paired-end reads of 150 bp length at ∼90× genome coverage. The sequence reads were deposited at the Sequence Read Archive (SRA) database (Bioproject accession number PRJNA999728). The Mutation Identification in Model Organism Genomes using Desktop PCs (MiModD) suite of bioinformatics tools (version 0.1.8; https://sourceforge.net/projects/mimodd/) was used on the Galaxy Europe bioinformatics server (https://usegalaxy.eu) to execute the ACCA variant mapping workflow as follows. The sequencing reads of the *cdaH-1* and wild-type F2 pools were aligned to the macronuclear reference genome of the wild-type strain SB210 (GenBank assembly accession GCA_000189635) (Eisen et al., 2006) and a multi-sample variant calling was conducted. Variants were annotated with predicted effect on gene products, and linkage scores were calculated on the basis of contrast between the allelic composition of the wild-type and the mutant pool. The linkage scores were plotted against the micronuclear genome assembly (GenBank assembly accession GCA_000261185.1) (Hamilton et al., 2016) coordinates along the five micronuclear chromosomes. This analysis displayed a peak in the ACCA signal at a location of ∼0.05 Mb of chromosome 5, and nearby variants with a predicted effect on a gene product were considered candidate causative variants.

### Phylogenetic and structural analyses

The predicted gene model for *CDAH/TTHERM_01345780* was corrected based on the mRNA sequence data available at the *Tetrahymena* Functional Genomics Database (http://tfgd.ihb.ac.cn), which revealed an additional intron near the end of the coding region. A phylogenetic analysis was performed using 497 sequences of human kinase domains (Modi and Dunbrack, 2019b) and the corresponding amino acid sequences of CdaH/TTHERM_01345780, TTHERM_00756520 and TTHERM_00726480. The following analyses were performed at the NGPhylogeny.fr server (Lemoine et al., 2019; https://ngphylogeny.fr/). A multiple sequence alignment was produced using the Multiple Alignment using Fast Fourier Transform (MAFFT) program (Katoh and Standley, 2013) and curated using trimAI (Capella-Gutiérrez et al., 2009). A neighbor-joining phylogeny was constructed using FastME and the statistical support of branches was tested by bootstrap resampling with 1000 replicates (Lefort et al., 2015). The tree was visualized with iTOL (https://itol.embl.de). A multiple sequence alignment of the entire amino acid sequences of CdaH and other Fused/Stk36 kinases was obtained with Clustal Omega at the European Molecular Biology Laboratory (EMBL)-European Bioinformatics Institute (EBI) (Sievers et al., 2011) and visualized with mView (Brown et al., 1998). Protein domains were detected using the SMART (http://smart.embl-heidelberg.de/) tool and homologs in other species were identified using the Blastp tool at the Cildb database (http://cildb.i2bc.paris-saclay.fr/).

### Genome editing in the macronucleus

To rescue the *cdaH-1* phenotype, starved IA450 cells were subjected to biolistic bombardment with a ∼1.5-kb fragment of *TTHERM_01345780* amplified from the wild-type genomic DNA (strain CU428) using the primers 5′-GCATTTCTCTAAAATTATTTTTGTATCC-3 and 5′-GTTCTGCAATTCCATTATCATC-3′. The bombarded (∼5×10^6^) and mock-transformed cells were cultured in SPPA medium at 25°C for 1 day, replicated onto fresh 96-well plates and incubated at 38°C. We searched for wells containing cells that regained the ability to multiply. To further confirm the location of the *cdaH-1* causal mutation, we constructed plasmids carrying a portion of the 5′ UTR of *TTHERM_01345780*, followed by *neo5,* the MTT1 promoter and a portion of the coding sequence of *TTHERM_01345780* that terminated either 63 bp upstream or 568 bp downstream of the suspected *cdaH-1* causal variant site. To construct the above plasmids, the 5′ UTR region was amplified with the primers 5′-GAATTGGAGCTCAATCTATAAAATCTTGATTTCTCTTT-3′ and 5′-TGCCATCCGCGGTCTAACAATCTAACATTAAAACA-3′and cloned between the SacI and SacII sites of pLF4gOEneo2-4 (Jiang et al., 2019b). The coding region of *TTHERM_01345780* was amplified with the forward primer 5′-ACTTAAAATAATGGCCAAGTCGACGATGGAGAATTATCATATTCTT-3′ and either 5′- AGGGAACAAAAGCTGGGTACCAAAATGTTTGTATTGTTT-3′ (long coding fragment) or 5′-AGGGAACAAAAGCTGGGTACCCAAAACCGAAATCGCATAATTTTACAA-3′ (short coding fragment), and cloned using the KpnI and SalI sites. The targeting fragments of the resulting plasmids (pMTT1_CDAHov_long and pMTT1_CDAHov_short) were separated from their plasmid backbones using KpnI and SacI and introduced into the *cdaH-1* mutants (IA450) by biolistic bombardment. Transformants were selected with 100 µg/ml paromomycin at room temperature. Wells containing transformants were replicated onto fresh SPPA medium with 400 µg/ml paromomycin, incubated overnight at 38°C and scored for cell multiplication.

To express CdaH–GFP, we added a DNA sequence encoding GFP, a 3′ UTR region of *BTU1* and a *neo5* selectable marker to the 3′ end of *TTHERM_01345780/CDAH* by homologous DNA recombination. The flanking DNA sequences for targeting *CDAH* were amplified using the following primer pairs: forward, 5′-GGGCGAATTGGCCGGCATTATTTCATTCAAAGTTTGTTATTTC-3′, and reverse, 5′-ATCAAGCTTGCCATCCGCGGTTATTGCATGTCCTAATC-3′; forward, 5′- GCTTATCGATACCGTCGACCACAAATTAATTTAAGAATTATGA-3′, and reverse: 5′- AGGGAACAAAAGCTGGGTACGATTATGTCGATTAAACTGAA-3′. The two fragments were subcloned into the plasmid pNeo2_4-GFP (Jiang et al., 2019b). The *CDAH* gene was tagged in the wild-type (CU428), *cdaA-1* (IA104) and *cdaI-1* (IA237) backgrounds. Transformants were selected with 100 µg/ml paromomycin. The copy number of the engineered allele was increased by the phenotypical assortment with increasing concentration of paromomycin. The targeting plasmids for GFP tagging of *CDAI* and *CDAA* in the *cdaH-1* background were described previously (Jiang et al., 2017, 2020).

### Microscopic imaging

*T. thermophila* cells were fixed and immunostained using the quick method that includes simultaneous fixation and/or permeabilization and drying of cells on the cover glass (Gaertig et al., 2013) with small modifications. After placing on the cover glass, 20 μl of cell culture was mixed with an equal volume of 0.25% Triton X-100 and 1% paraformaldehyde in PHEM buffer (60 mM PIPES, 25 mM HEPES, 10 mM EGTA and 2 mM MgSO_4_, pH 6.9). The cover glass was air dried, washed three times with PBS and incubated with primary antibodies in PBS supplemented with 3% BSA fraction V and 0.01% Tween-20. The primary antibodies were: polyclonal rabbit anti-GFP (Rockland Immunochemicals, 600-401-215; 1:800), monoclonal mouse anti-centrin 20H5 (EMD Millipore, 41624; ∼1:200–1:400), and anti-polyglycine polyclonal serum 2302 (Shang et al., 2002) (1:200). The secondary antibodies were conjugated to either Cy3 or FITC (Jackson ImmunoResearch, 115-095-146 and 111-165-003; 1:100–1:300). The nuclei were stained with DAPI (Sigma-Aldrich). The stained cells were embedded in 90% glycerol, 10% PBS supplemented with 100 mg/ml DABCO (Sigma-Aldrich). Confocal images were collected on a Zeiss LSM 710 microscope with a Plan-Apochromat 63×/1.40 oil DIC M27 objective. SR-SIM imaging was conducted on an ELYRA S1 microscope equipped with a 63× NA 1.4 Oil Plan-Apochromat DIC objective. The optical slices were processed using Fiji/ImageJ (*z*-project tool). To image actin and CdaH–GFP, the CdaH–GFP-expressing cells were fixed with cold methanol (−80°C) for 30 min, washed three times with 1 ml of PBS, followed by a wash with 1 ml of PBS containing 1% BSA (PBSB) for 30 min. The primary and the secondary antibodies used were the anti-*Tetrahymena* Act1 actin rabbit serum (Shiozaki et al., 2013; 1:200 dilution in PBSB) and the goat anti-rabbit IgG H/L antibodies coupled to Alexa Fluor 594 (Thermo Fisher Scientific, R37117; 1:200), respectively. The cells were observed using an Olympus BX51 fluorescence microscope equipped with a 100× lens (NA 1.40) and the images were collected on a Hamamatsu Photonics ORCA-3CCD camera. For SR-SIM co-imaging of CdaH–GFP and actin, cells were fixed and dried on coverslips as described above, the coverslips washed three times for 5 min in PBS, treated with 1% SDS in PBS for 20 min, and washed three times for 5 min in PBS. The primary antibodies used were anti-Act1 guinea pig serum (Shiozaki et al., 2013; 1:80) and anti-GFP (Rockland Immunochemicals, 600-401-215; 1:800), and the secondary antibodies used were goat-anti-guinea pig IgG-FITC (Sigma-Aldrich, F6261; 1:100) and goat-anti-rabbit IgG-Cy3 (Jackson ImmunoResearch, 111-165-003; 1:200). The anti-actin antibodies were validated based on detection of a single band with the size expected for actin on western blots using total extracts of *T. thermophila.* The TIRF microscope set-up has been previously described (Lechtreck, 2016). TIRF imaging was done as previously described (Jiang et al., 2015) except that cells were immobilized by entrapment in a small volume and without NiCl_2_. To perform FRAP, a 488 nm laser beam was split using a zero-order half-wave plate and a broad band polarized beam splitter. One of the beams was used for TIRF illumination and the other beam was send to the specimen for photobleaching. The bleaching beam was expanded using a 3× beam expander, focused using a 200 mm plano-convex lens and a 35 mm plano-convex lens and recombined with the TIRF beam using a polarized beam splitter (all parts from Thorlabs Inc.). A motorized mirror connected to a joystick (Newfocus) was used to move the bleaching laser to the desired location and the size of the laser spot was controlled manually by moving the 35 mm lens. Samples were prepared for electron microscopy as previously described (Vasudevan et al., 2015).

### Statistical analysis

The sample sizes were chosen based on the previous research. To quantify the phenotypic defects, 10–50 dividing cells were scored per genotype. Differences were evaluated by GraphPad PRISM software. An unpaired two-tailed *t*-test was used and *P*<0.05 was considered to be statistically significant.

## Supplementary Material

10.1242/joces.261256_sup1 Supplementary information
