# Supplementary material for: Global and local functions of the Fused kinase ortholog CdaH in intracellular patterning in Tetrahymena
Source: J Cell Sci. 2023 Oct 4;137(5):jcs261256. doi: 10.1242/jcs.261256 (PMC10565251; doi:10.1242/jcs.261256)
Supplement: Supplementary information [file joces-137-261256-s1.pdf]

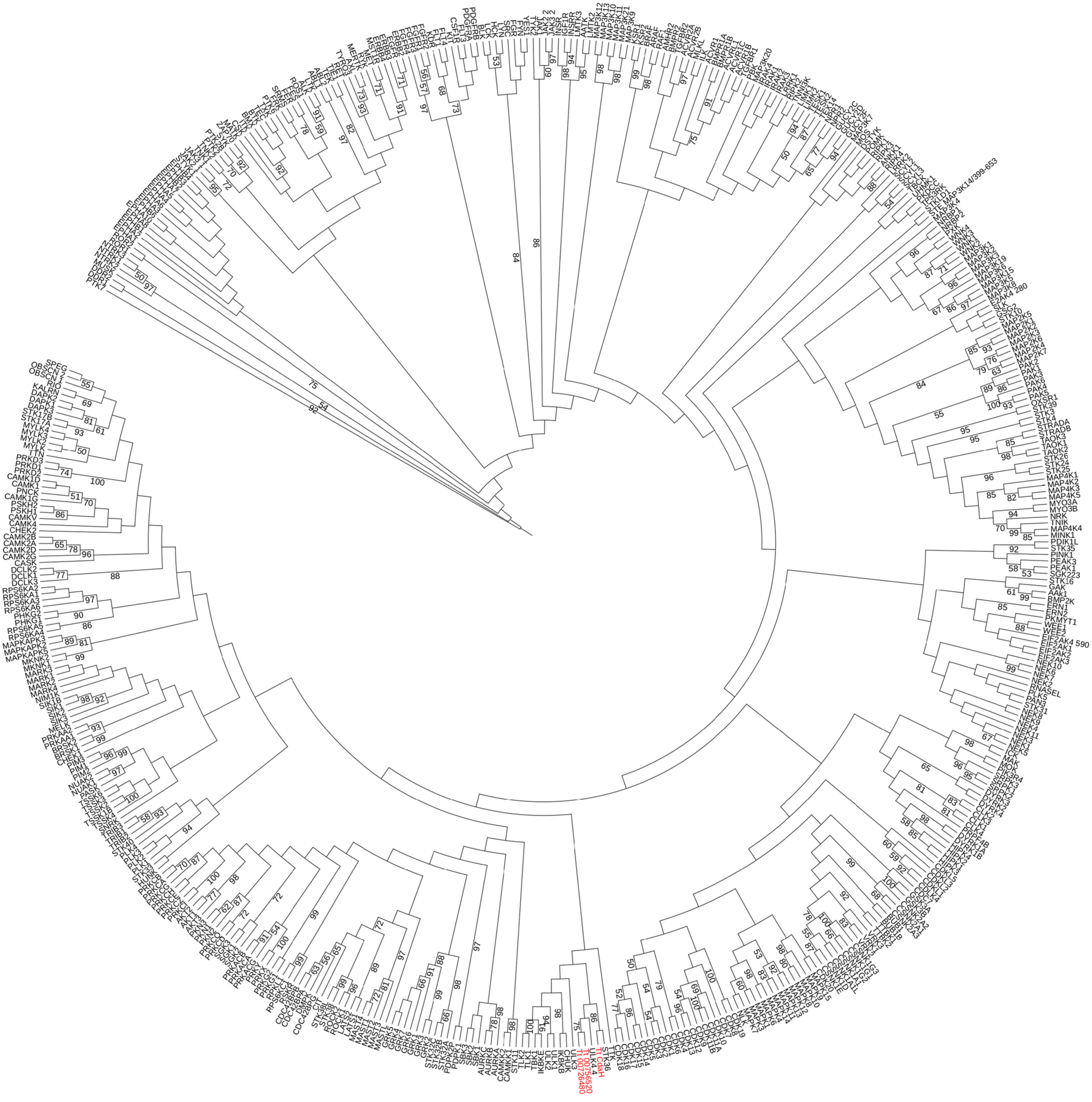

Fig. S1. CdaH as an STK36 ortholog. A neighbor-joining phylogenetic analysis was performed using 497 sequences of human kinase domains (Modi and Dunbrack, 2019b) and kinase domain sequences of Tetrahymena: CdaH/ TTHERM_01345780, TTHERM_00756520 and TTHERM_00726480. The numbers represent statistical support for branches based on 1000 bootstrap resamples. The tree is rooted to the human PLK7. The three Tetrahymena sequences are labeled in red.

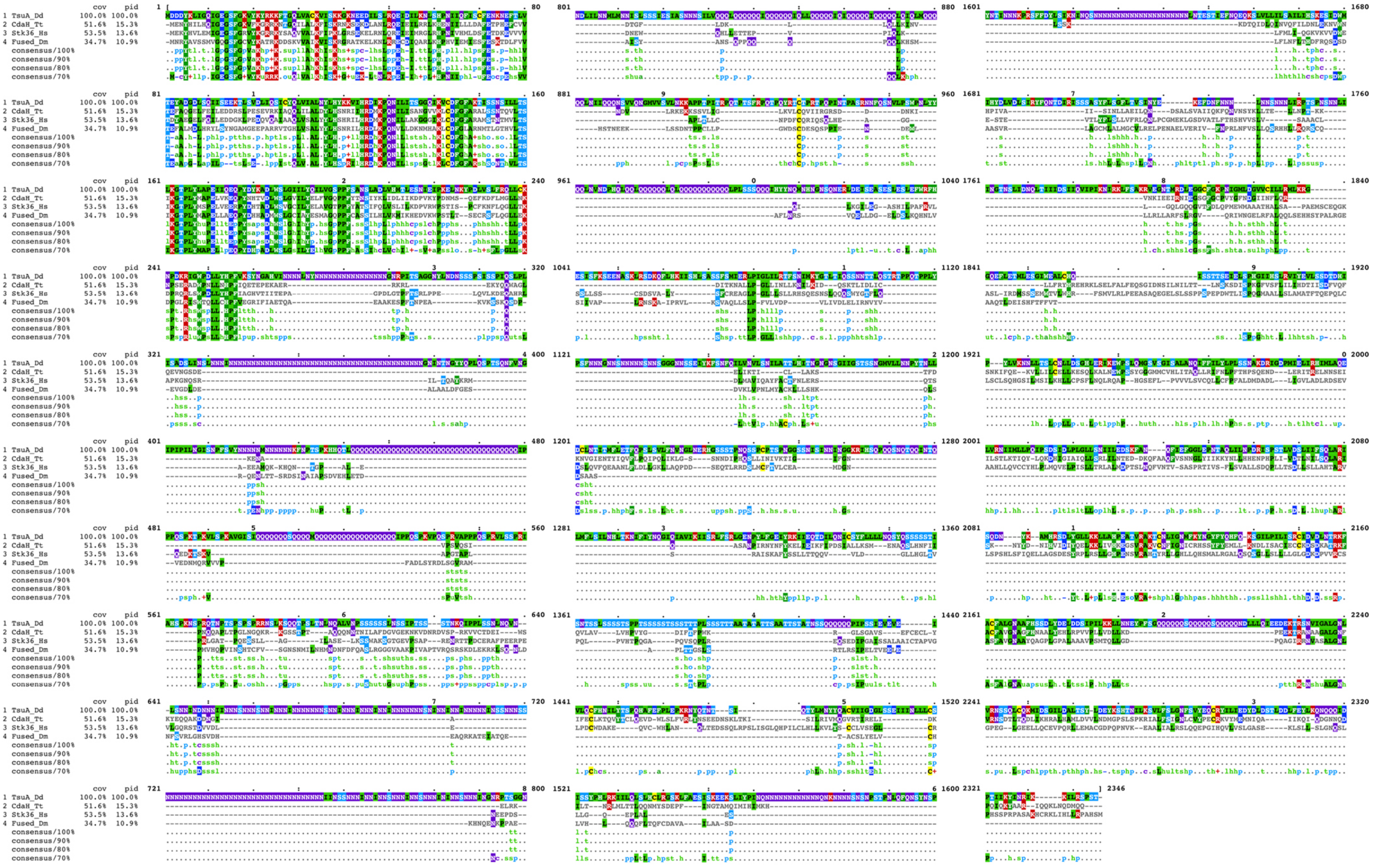

Fig. S2. A multiple amino acid sequence alignment of CdaH and Fused/Stk36 orthologs. A multiple sequence alignment of CdaH, human Stk36, Fu of *D. melanogaster* and TsuA of *D. discoideum.* Note strong homology within the N-terminal kinase domain and a weaker homology outside of the kinase domains.

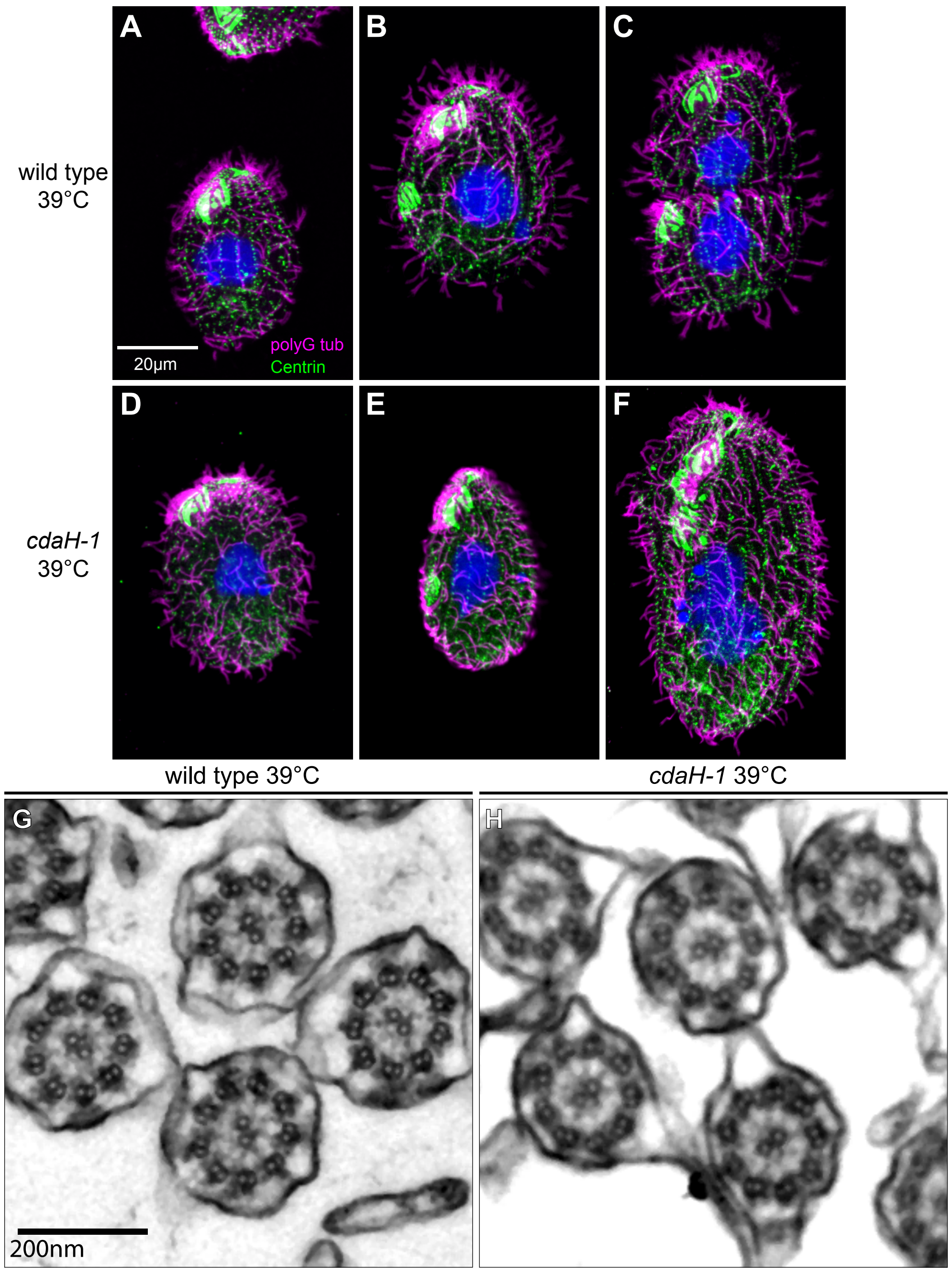

Fig. S3. *cdaH-1* mutants assemble structurally normal 9+2 cilia.
(A-F) Confocal images of the wild-type and *cdaH-1* mutant cells labeled by the anti-polyG tubulin antibodies (magenta), the anti-centrin antibody (green) and DAPI (blue). (G,H) Transmission electron micrographs of sections of the wild-type and the *cdaH-1* mutant cell with cross-sections of oral cilia. The images are representative. For the mutant, we analyzed a total 83 cross-sections of axonemes on multiple sections representing multiple cells.

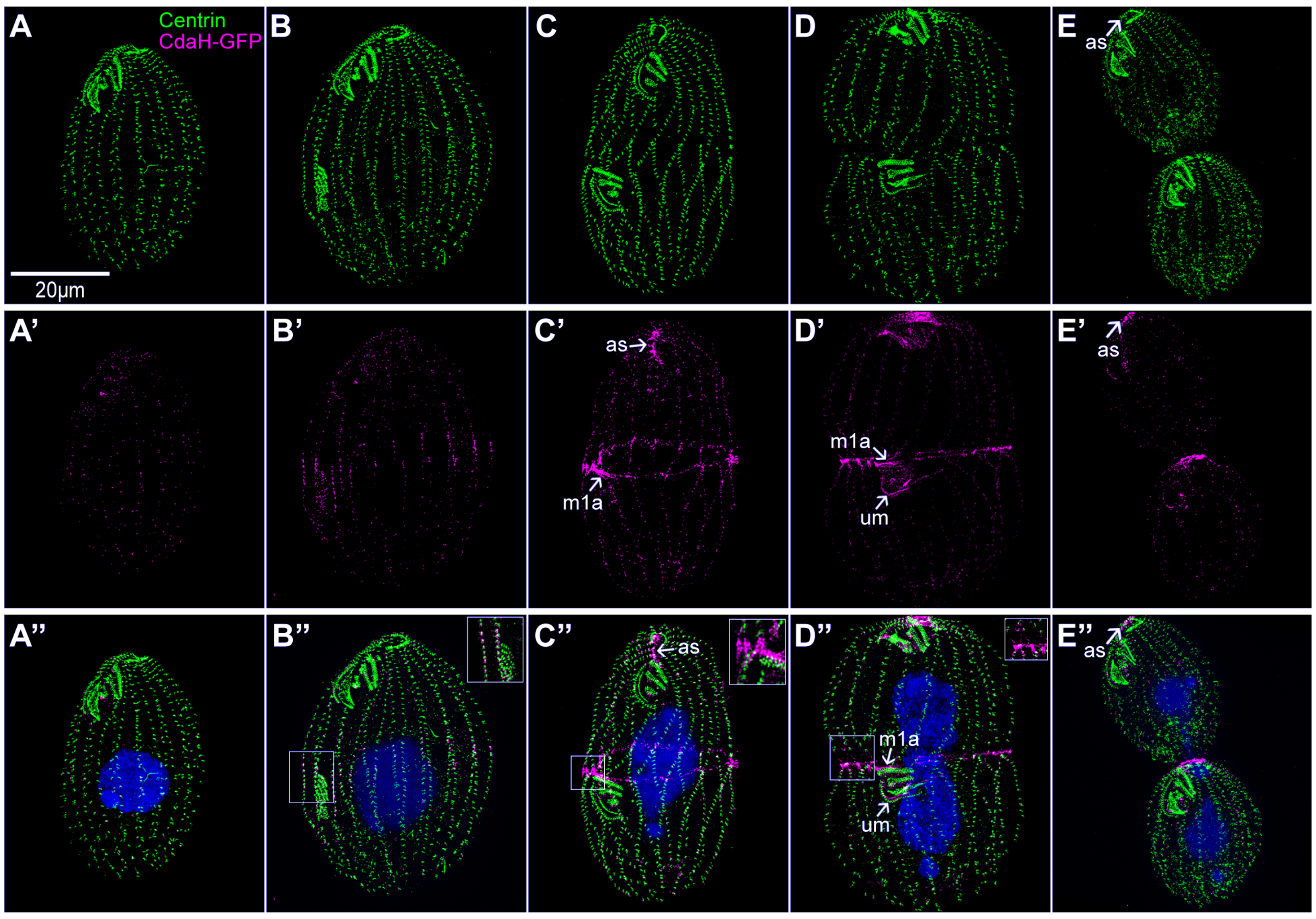

Fig. S4. SR-SIM imaging of CdaH-GFP. (A-E''). The CdaH-GFP expressing cells were labeled by immunofluorescence using the anti-GFP antibodies (magenta), the anti-centrin antibody (green) and DAPI (blue). (A-A'') Interphase. (B-B") Early OP development. (C-C") Cortical subdivision.

(D-E") Cytokinesis. Abbreviations: as, anterior suture; m1a, M1 arc; um, undulating membrane. The imaging of CdaH-GFP was conducted 5 times and representative images are displayed.

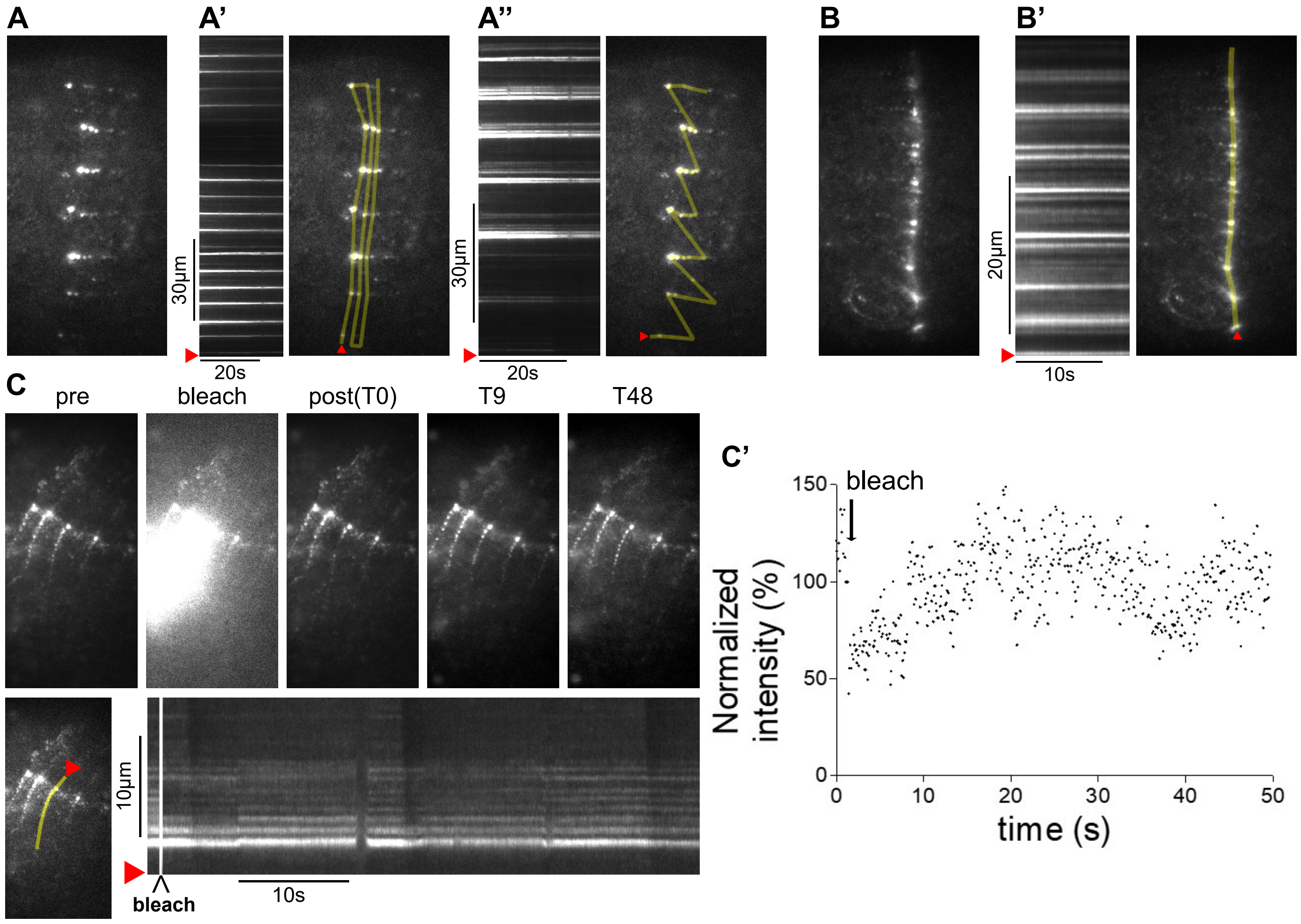

Fig. S5. TIRF imaging of CdaH-GFP. (A-B'). Kymograms reveal that the dot signals are stationary and there is no detectable movement of CdaH-GFP between the dots, both along the same streak and between the streaks. The yellow lines mark the areas analyzed in the kymograms. The red arrowheads orient the kymograms. (C-C') FRAP experiment reveals that CdaH-GFP within the streak turns over rapidly. Following bleaching with a focused laser beam the signal intensities were analyzed using kymograms. Individual frame for the image showing bleach, and 10 frame average for the rest of the images.
The quantitative analysis of the signal intensity in the course of FRAP is shown in C'.
The data are representative of observations done on 5 cells in the posterior streaks stage.
The live imaging experiments were conducted twice and representative images are shown.

Journal of Cell Science • Supplementary information

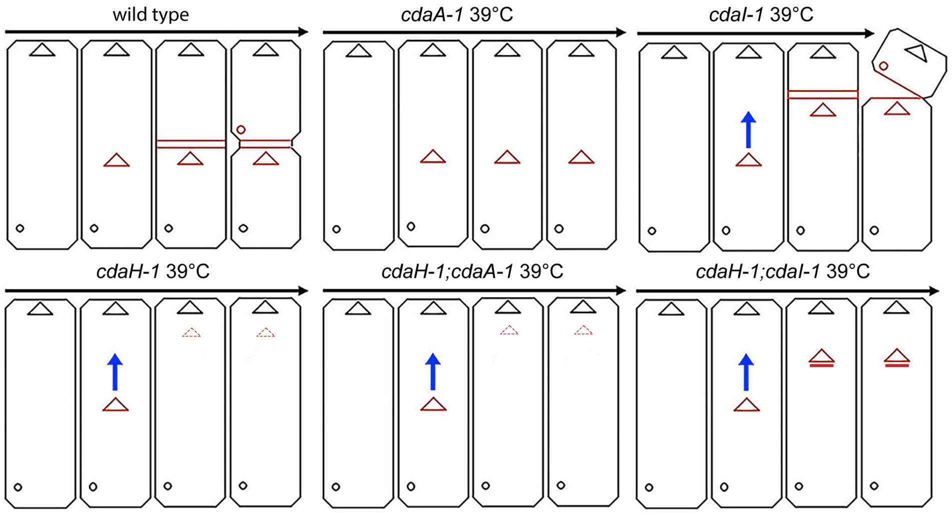

Fig. S6. A comparison of the phenotypes of single and double mutants used in this study. The following cortical structures are shown in black: oral apparatus (triangle), division boundary (horizontal bar), and contractile vacuole pore (circle). Using the same shapes, the new structures that form during cell division are shown in red. The blue arrows show the direction of displacement of the OP. The dashed line depicts OP undergoing degradation.

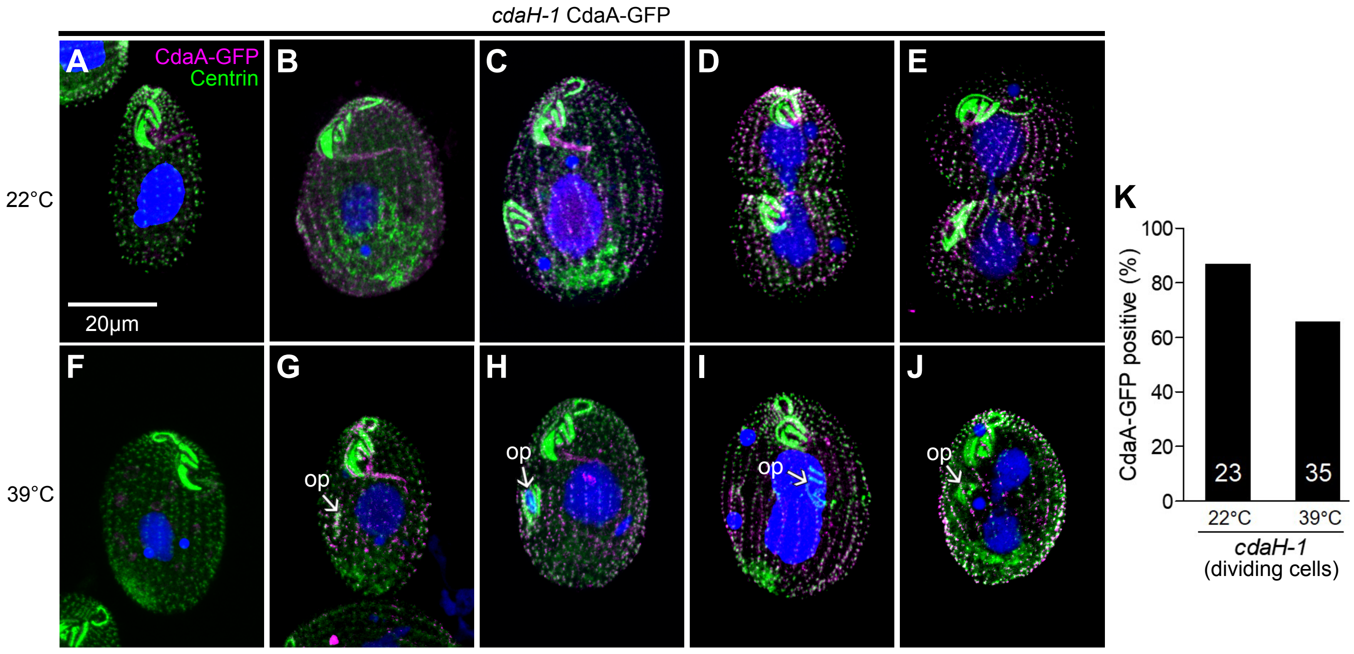

Fig. S7. *cdaH-1* expression does not affect the cortical distribution of CdaA.
(A-J) Confocal images of *cdaH-1* cells expressing CdaA-GFP. The cells were labeled by the anti-GFP antibodies (magenta), anti-centrin (green) antibody and DAPI (blue). Before staining, the cells were incubated at either 22°C (A-E) or 39°C (F-J) for 2 hr. (K) The graph shows the fractions of (stage appropriate) CdaA-GFP-positive dividing cells in the *cdaH-1* mutants. The representative images are derived from 3 independent experiments.

Journal of Cell Science • Supplementary information

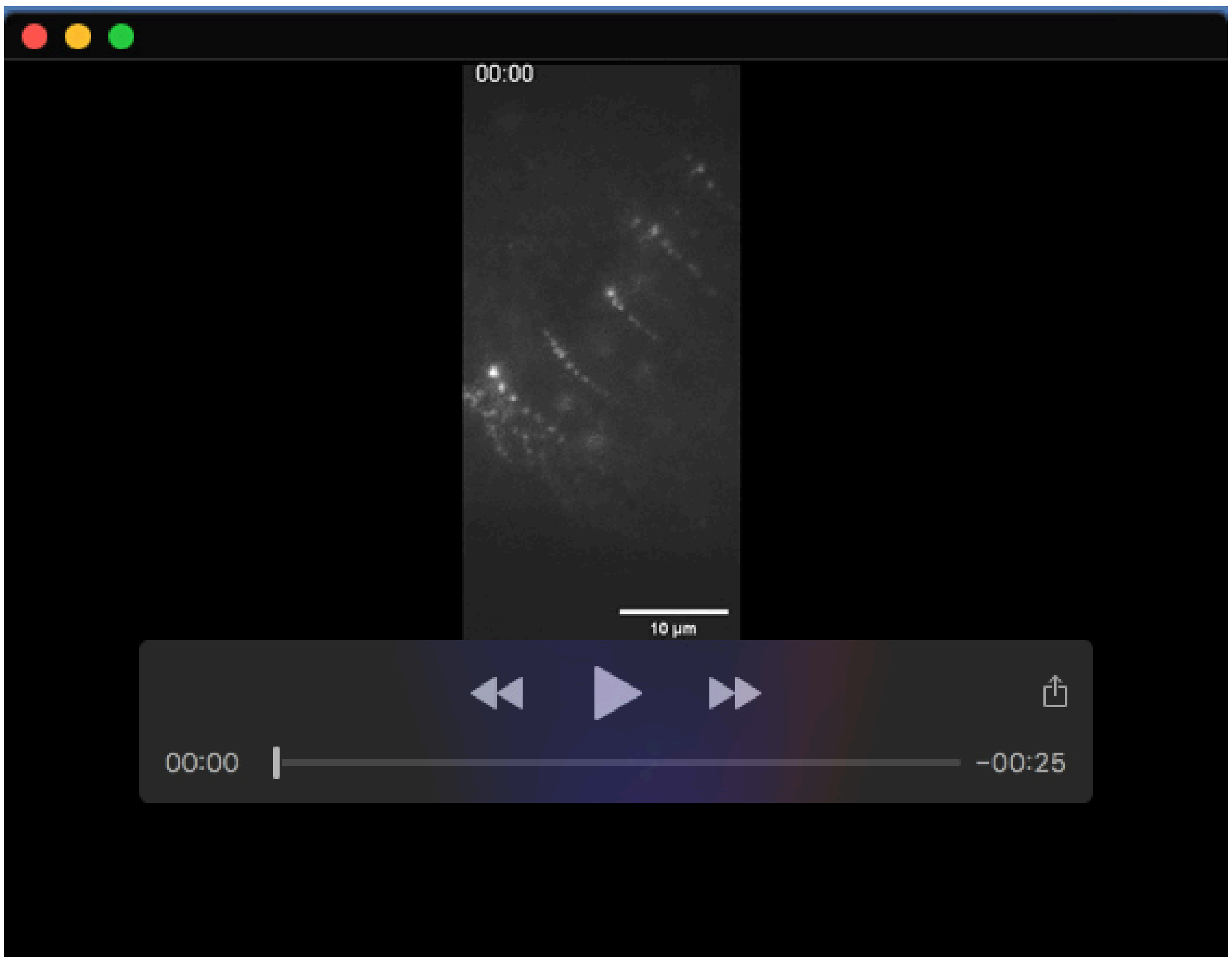

Movie 1. A FRAP TIRF imaging of the CDAH-GFP expressing cell at the stage of "posterior streaks" prior to the cortical subdivision.

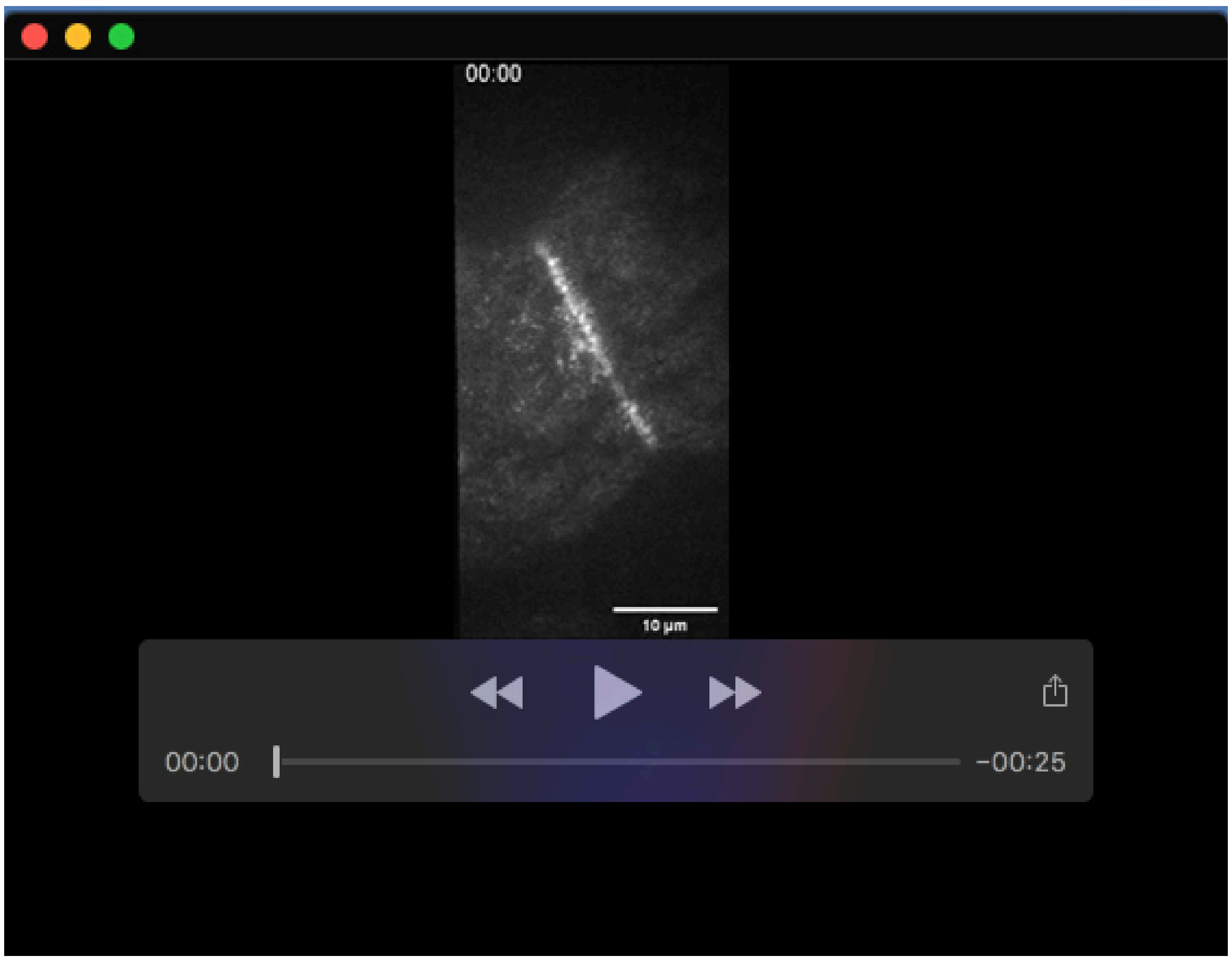

Movie 2. A FRAP TIRF experiment showing a rapid turnover of CdaH-GFP in the subequatorial ring.

Journal of Cell Science • Supplementary information
