# Supplementary material for: Global and local functions of the Fused kinase ortholog CdaH in intracellular patterning in Tetrahymena
Source: J Cell Sci. 2023 Oct 4;137(5):jcs261256. doi: 10.1242/jcs.261256 (PMC10565251; doi:10.1242/jcs.261256)
Supplement: Supplementary file 1 [file joces-137-261256_review_history.pdf]

Journal of Cell Science

Global and local functions of the Fused kinase ortholog CdaH in intracellular patterning in *Tetrahymena*

Chinkyu Lee, Wolfgang Maier, Yu-Yang Jiang, Kentaro Nakano, Karl F. Lechtreck and Jacek Gaertig
DOI: 10.1242/jcs.261256

Editor: David Bryant

Review timeline

Original submission:	19 April 2023
Editorial decision:	2 June 2023
First revision received:	3 August 2023
Accepted:	29 August 2023

Original submission

First decision letter

MS ID#: JOCES/2023/261256

MS TITLE: Global and local functions of a Fused/Stk36 kinase, CdaH, in intracellular patterning in Tetrahymena

AUTHORS: Chinkyu Lee, Wolfgang Maier, Yu-Yang Jiang, Kentaro Nakano, Karl F Lechtreck, and Jacek Gaertig
ARTICLE TYPE: Research Article

We have now reached a decision on the above manuscript. To see the reviewers' reports and a copy of this decision letter, please go to: https://submit-jcs.biologists.org and click on the 'Manuscripts with Decisions' queue in the Author Area. (Corresponding author only has access to reviews.)

As you will see, all reviewers gave favourable reports but raised some critical points that will require amendments to your manuscript. I hope that you will be able to carry these out as the majority of these are text or figure editing or further explanation. I would like to be able to accept your paper, depending on further comments from reviewers. I note that Reviewer 2 asks for knockout or knockdown, but I do acknowledge (as does another reviewer in their comments to me) that this can be considerable work in Tetrahymena. If you are able to do this validation in reasonable time I encourage it, but I am also open to explanation of where this is technically or time-wise not possible. Such a scenario would need to be fully justified to the reviewers.

Please ensure that you clearly highlight all changes made in the revised manuscript. Please avoid using 'Tracked changes' in Word files as these are lost in PDF conversion.

I should be grateful if you would also provide a point-by-point response detailing how you have dealt with the points raised by the reviewers in the 'Response to Reviewers' box. Please attend to all of the reviewers' comments. If you do not agree with any of their criticisms or suggestions please explain clearly why this is so.

© 2023. Published by The Company of Biologists under the terms of the Creative Commons Attribution License (https://creativecommons.org/licenses/by/4.0/).

Reviewer 1

Advance summary and potential significance to field

The manuscript entitled "Global and local functions of a Fused/Stk36 kinase CdaH, in intracellular patterning in Tetrahymena" from Chinkyu Lee, Wolfgang Maier, Yu-Yang Jiang, Kentaro Nakano, Karl F. Lechtreck, and Jacek Gaertig describes the molecular identification and characterization of the Tetrahymena mutated cdaH-1 allele previously described by Frankel and coworkers (1980). In the present work the authors by using comparative whole genome sequencing show that the CDAH gene in Tetrahymena genome (TTHERM_01345780 gene) encodes an ortholog of highly conserved Fused/Stk36 kinases. This group of kinases in animal cells are involved in the hedgehog signaling during embryonic development. The demonstration that cdaH-1 allele encodes a mutant Fused/Stk36 kinase is conclusive since the cell division-blockage of the cdaH-1 cells is only rescued, by homologous DNA recombination, by a wild-type genomic fragment spanning the variant position in TTHERM_01345780. Additionally, the sequencing of the TTHERM_01345780 in independent rescues showed the presence of the wild type and variant sequence consistent with partial replacement of the 90 gene copies in the macronucleus. Regarding the characterized phenotypes the authors proposed that CdaH is an important molecule in defining the Tetrahymena intracellular patterning. Mainly in the ciliate CdaH has multiple functions global or local, namely: i) maintaining the position of the oral apparatus relatively to the anterior-posterior cell polarity axis; and in the accomplishment of cortical subdivision and cytokinesis, as well as in the morphogenesis of the oral apparatus, respectively. Besides a few concerns, the results are solid and have the potentiality to give new clues about the Fused/Stk36 kinases roles in metazoan development by revealing core mechanisms and how they operate inside the ciliate single complex cell. Ciliates are good models to highlight regulatory steps that are probably diluted in the complex regulation of cells that live constrained by multiple cell-cell interactions and specific extracellular matrix environments. In conclusion, the manuscript deserves to be published.

Comments for the author

Besides a few concerns, the results are solid and have the potentiality to give new clues about the Fused/Stk36 kinases roles in metazoan development by revealing core mechanisms and how they operate inside the ciliate single complex cell. Ciliates are good models to highlight regulatory steps that are probably diluted in the complex regulation of cells that live constrained by multiple cell-cell interactions and specific extracellular matrix environments. In conclusion, the manuscript deserves to be published.

A few concerns:
1- The authors refer that "No CdaH-GFP signal above the background was present in nondividing cells (Fig. 4A,A'). " Is this really true for the old oral apparatus (Figure 4A and A´)? Please clarify and discuss.

2- The authors refer that "after photobleaching, the dot signals recovered to near pre-bleach level within a few seconds (Fig. S3C-C'), indicating a rapid turnover of the dot associated CdaH, likely by exchange with soluble CdaH in the cell body". This rapid turnover is dependent on the more dynamic intracytoplasmic microtubules?

3- It is referred that "These two CdaH-GFP pools (ring and AS) could be related, as both are positioned near the anterior ends of ciliary rows (old and new, respectively)." Could the authors better clarify how they envisage the relationship between the ring and AS? Is merely in positioning? Is AS required for the final position of the old and new oral apparatus?

4- "The CdaH ring is visible shortly before the actin ring, suggesting that CdaH or its complex polymerizes". Is the word polymerizes correctly used in this sentence? The authors have evidence for this? If yes they should support this idea that is also resumed in the last sentence of the discussion.

© 2023. Published by The Company of Biologists under the terms of the Creative Commons Attribution License (https://creativecommons.org/licenses/by/4.0/).

Reviewer 2

Advance summary and potential significance to field

In this manuscript entitled “Global and local functions of a Fused/Stk36 kinase, CdaH, in intracellular patterning in Tetrahymena”, Lee and colleagues analyses a thermosensitive mutant CdaH-1 in Tetrahymena. This paper is the third paper of a series of Tetrahymena mutant analysis (Jiang et al, 2017 and Jiang et al, 2020). As previously, the paper characterized the phenotype of the mutant at the restrictive temperature, identified the mutated gene with its mutated amino-acid, and localized the protein as a GFP-fusion protein. Because the phenotypes of this mutant show similarities to the two previously identified mutants (Jiang et al, 2017 and Jiang et al, 2020), the authors investigates epistatic relationships between the three mutants.

The interesting part of the paper is that CdaH encodes a Fused/Stk36 protein, an evolutionary conserved kinase, although it plays distinct functions in different species. The observed mutation in the kinase domain appears to reduce kinase activity, but it has not been demonstrated. Formation of the contractile ring required for cytokinesis is absent in this mutant and the new oral apparatus (OP) migrates anteriorly. The fusion protein CdaH-GFP, localizes at multiple sites. In early dividing cells both the OP and dots along ciliary rows, which could correspond to basal bodies are decorated. But interestingly, before cytokinesis, the dots along the ciliary rows in the hemi-posterior cells increase in intensity before forming a ring. This ring forms slightly before the actin ring and the two rings colocalize during the process of cell constriction.
While interesting for the Tetrahymena community the paper is mostly descriptive.

Comments for the author

1) It seems that the CdaH protein relocates from the BB dots in the hemi-posterior cell near the boundary division to a ring. How do the authors imagine this remodelling to occur?
2) The mutation in the mutant is in the kinase domain suggesting that the kinase activity is most probably reduced. However, the protein is still present. I wonder if the results would be easier to interpret with a knock-out or knock-down of the protein? The authors used kinase deficient mutants to perform their epistatic experiment. They say “loss of function alleles” p 11. But then, they suggest “that CdaI activity may inhibit CdaH activity that prevent the OP degradation”p12 and later on “CdaH stimulating the CdaI activity…”. This is quite confusing. Can the authors perform again these experiments with at least one knock-down or knock-out mutant?
3) The CdaH protein seems closer to the human Stk36 protein, but the function seems different. An alignment between the Tetrahymena protein and other Fused protein found during evolution will be necessary for the reader to better interpret the results?
4) The authors mention an other Fused-like protein in the Tetrahymena genome (TTHERM_726480), which appears to be closer to the mammalian ULK4. Could this protein have redundancy with the CdaH one? Similarly, an alignment would be necessary.
5) Regarding the lack of defects in the axonemal structure in the CdaH-1 mutant: It was observed previously in one PCD-affected individual with situs solitus with a loss-of-function mutation in Stk36 gene, that the axonemal structure lacked the central pair. I wonder if the normal axonemal structure is because the electron microscopy was done randomly and not on cells at the onset of cytokinesis (Figure1 stage 3)? Have the basal bodies of the posterior hemi-cell, showing the most significant staining, be analyzed.

Minor comments
1) The temperature vary in the experiments between 38° and 39°. Why? Is it a writing mistake?
2) Figure 4: it is disturbing to see the the CdaH-GFP signal in red while the centrin dot are in green. Is it some reasons for this choice? Can the authors put the GFP signal in green and the centrin signal in red? Same for Figure 6 G-G,’H-H’, Figure 7, S2 and S5.
3) Electron microscope method is absent.

© 2023. Published by The Company of Biologists under the terms of the Creative Commons Attribution License (https://creativecommons.org/licenses/by/4.0/).

Reviewer 3

Advance summary and potential significance to field

Lee et al. describe the identification of the causal gene for a Tetrahymena patterning mutant cdaH as a Fused/Stk36 kinase. Furthermore, they go on to describe the localization of CdaH throughout the cell division cycle and characterize genetic interactions with other known patterning genes, cdaA and cdaI. The authors implicate cdaH in working to position the division plane appearing even before actin to the site of division, in a CdaA/Cyclin E-dependent process. CdaH also seems to interact with CdaI to control the formation of membranelles during OA patterning. Overall, I think this is a very interesting paper that adds to the growing number of patterning genes that appear to be from highly conserved pathways throughout Eukaryotes. These findings represent a glimpse into different evolutionary specializations of these genes, or potentially some of their ancestral functions. I recommend this manuscript for publication after minor revisions.

Comments for the author

Major Comments:
1. On page 6 the authors discuss mapping the causative gene and rescuing the phenotypes by inserting functional copies of either of the two closely linked genes. They say that TTHERM_00649260 were not observed to rescue the mutant phenotype, but these data don't appear to be shown in Figure 3. All of the rescues shown, and discussed in the figure legend, are rescues of TTHERM_01345780.

2. There are a few places the authors should list the N for experiments where representative images are shown. For example, in Figure 5 the authors show kymogram and FRAP data but it is unclear how many cells were imaged to make these conclusions.

3. On page 12, referring to data in Figure 8, the authors are making claim that there is a significant enhancement of the "excessive number of membranelles" phenotype in the cdaH-1;cdaI-1 double mutants as compared to cdaI-1 alone. It seems the authors are referring to an increased penetrance of the phenotype in the double, rather than an enhancement of the phenotype where one might predict more membranellar bands to form. I think the language could be made clearer here, but if there is an increase in the number of membranellar bands seen in their data (which might be the case in the representative images), that would be an interesting thing to quantify.

4. Also referring to the data in Figure 8J, the authors should perform an appropriate statistical test to demonstrate a significant increase in the % of cells displaying the "Additional membranelle" phenotype in the double-mutant as compared to the cdaI-1 single mutant.

Minor Comments:
1. Authors should avoid the use of Red and Green colors together, or entirely which would increase accessibility to colorblind readers. Good options are described in an ASCB article (https://www.ascb.org/science-news/how-to-make-scientific-figures-accessible-to-readers-with-color-blindness/)

2. Color choices in the figures could be more consistent. For example, sometimes actin is shown red, other times it is green. In Figure 6 Actin is shown as red in panels A-E" but is green in panels G-H". While the figure is clearly labeled consistent colors would help to avoid reader confusion.

3. Movie 1 and Movie 2 should have time-stamps and scale bars

4. On page 4, there is a typo where "sculpturing" should probably be "sculpting"

5. Figure 6 Legend: Typo, "labeled with polyclonal anti-Act1 actin antibodies (red0…" where the 0 should be a).

© 2023. Published by The Company of Biologists under the terms of the Creative Commons Attribution License (https://creativecommons.org/licenses/by/4.0/).

First revision

Author response to reviewers' comments

We would like to thank the reviewers for their time, constructive criticisms and excellent suggestions. Below there are our responses and the revisions made.

Reviewer 1 Advance Summary and Potential Significance to Field:
The manuscript entitled "Global and local functions of a Fused/Stk36 kinase, CdaH, in intracellular patterning in Tetrahymena" from Chinkyu Lee, Wolfgang Maier, Yu-Yang Jiang, Kentaro Nakano, Karl F. Lechtreck, and Jacek Gaertig describes the molecular identification and characterization of the Tetrahymena mutated cdaH-1 allele previously described by Frankel and coworkers (1980). In the present work the authors by using comparative whole genome sequencing show that the CDAH gene in Tetrahymena genome (TTHERM_01345780 gene) encodes an ortholog of highly conserved Fused/Stk36 kinases. This group of kinases in animal cells are involved in the hedgehog signaling during embryonic development. The demonstration that cdaH-1 allele encodes a mutant Fused/Stk36 kinase is conclusive since the cell division-blockage of the cdaH-1 cells is only rescued, by homologous DNA recombination, by a wild-type genomic fragment spanning the variant position in TTHERM_01345780. Additionally, the sequencing of the TTHERM_01345780 in independent rescues showed the presence of the wild type and variant sequence consistent with partial replacement of the 90 gene copies in the macronucleus. Regarding the characterized phenotypes the authors proposed that CdaH is an important molecule in defining the Tetrahymena intracellular patterning. Mainly in the ciliate CdaH has multiple functions global or local, namely: i) maintaining the position of the oral apparatus relatively to the anterior-posterior cell polarity axis; and in the accomplishment of cortical subdivision and cytokinesis, as well as in the morphogenesis of the oral apparatus, respectively.
Besides a few concerns, the results are solid and have the potentiality to give new clues about the Fused/Stk36 kinases roles in metazoan development by revealing core mechanisms and how they operate inside the ciliate single complex cell. Ciliates are good models to highlight regulatory steps that are probably diluted in the complex regulation of cells that live constrained by multiple cell-cell interactions and specific extracellular matrix environments. In conclusion, the manuscript deserves to be published.

Reviewer 1 Comments for the Author:
Besides a few concerns, the results are solid and have the potentiality to give new clues about the Fused/Stk36 kinases roles in metazoan development by revealing core mechanisms and how they operate inside the ciliate single complex cell. Ciliates are good models to highlight regulatory steps that are probably diluted in the complex regulation of cells that live constrained by multiple cell-cell interactions and specific extracellular matrix environments. In conclusion, the manuscript deserves to be published.

A few concerns:
1- The authors refer that "No CdaH-GFP signal above the background was present in nondividing cells (Fig. 4A,A'). " Is this really true for the old oral apparatus (Figure 4A and A´)? Please clarify and discuss.

Parts of the oral apparatus especially its posterior region, are commonly labeled non-specifically by secondary antibodies. Somatic basal bodies are also weakly labeled non-specifically (for example, see the negative control in figure 6E in Jiang et al 2017). We added a clarification to the legend of figure 4A).

2- The authors refer that "aker photobleaching, the dot signals recovered to near pre-bleach level within a few seconds (Fig. S3C-C'), indicating a rapid turnover of the dot associated CdaH, likely by exchange with soluble CdaH in the cell body". This rapid turnover is dependent on the more dynamic intracytoplasmic microtubules?

We revised the manuscript to mention the possibility that intracytoplasmic microtubules mediate

© 2023. Published by The Company of Biologists under the terms of the Creative Commons Attribution License (https://creativecommons.org/licenses/by/4.0/).

rapid loading of CdaH-GFP to the cortex.

3- It is referred that “These two CdaH-GFP pools (ring and AS) could be related, as both are positioned near the anterior ends of ciliary rows (old and new, respectively).” Could the authors better clarify how they envisage the relationship between the ring and AS? Is merely in positioning? Is AS required for the final position of the old and new oral apparatus?

We intended to point out that the two CdaH subpools share properties: 1) appear at about the same time during cell division and 2) localize near the anterior ends of ciliary rows (corresponding to the plus ends of longitudinal microtubules). While the ring pool correlates with the spatially-proximal phenotypic defects in cortical subdivision and cytokinesis, currently no defects can be associated with the AS pool. The reviewer exposes an important limitation of our study: we merely correlate the specific phenotypic defects with the most proximal pools of CdaH. In the future, an important goal will be to identify potential interactors that are required for targeting or execution of CdaH functions at specific locations.

4- “The CdaH ring is visible shortly before the actin ring, suggesting that CdaH or its complex polymerizes”. Is the word polymerizes correctly used in this sentence? The authors have evidence for this? If yes they should support this idea that is also resumed in the last sentence of the discussion.

We agree that the term “polymerizes” is not proper as it not known whether the ring formation is driven by CdaH-CdaH interactions. CdaH may associate with another protein that itself polymerizes (e.g. actin). Accordingly, we revised the Discussion section.

Reviewer 2 Advance Summary and Potential Significance to Field:
In this manuscript entitled “Global and local functions of a Fused/Stk36 kinase, CdaH, in intracellular patterning in Tetrahymena”, Lee and colleagues analyses a thermosensitive mutant CdaH-1 in Tetrahymena. This paper is the third paper of a series of Tetrahymena mutant analysis (Jiang et al, 2017 and Jiang et al, 2020). As previously, the paper characterized the phenotype of the mutant at the restrictive temperature, identified the mutated gene with its mutated amino-acid, and localized the protein as a GFP-fusion protein. Because the phenotypes of this mutant show similarities to the two previously identified mutants (Jiang et al, 2017 and Jiang et al, 2020), the authors investigates epistatic relationships between the three mutants. The interesting part of the paper is that CdaH encodes a Fused/Stk36 protein, an evolutionary conserved kinase, although it plays distinct functions in different species. The observed mutation in the kinase domain appears to reduce kinase activity, but it has not been demonstrated.

Formation of the contractile ring required for cytokinesis is absent in this mutant and the new oral apparatus (OP) migrates anteriorly. The fusion protein CdaH-GFP, localizes at multiple sites. In early dividing cells both the OP and dots along ciliary rows, which could correspond to basal bodies are decorated. But interestingly, before cytokinesis, the dots along the ciliary rows in the hemi-posterior cells increase in intensity before forming a ring. This ring forms slightly before the actin ring and the two rings colocalize during the process of cell constriction.
While interesting for the Tetrahymena community the paper is mostly descriptive.

Reviewer 2 Comments for the Author:
1) It seems that the CdaH protein relocates from the BB dots in the hemi-posterior cell near the boundary division to a ring. How do the authors imagine this remodelling to occur?

It is certainly possible that CdaH relocates from the posterior streaks to the ring, but we think that given the high rate of turnover it is perhaps more likely that the two pools are not directly linked. Future studies could use a photoconvertible CdaH to examine the flows of CdaH.

2) The mutation in the mutant is in the kinase domain suggesting that the kinase activity is most probably reduced. However, the protein is still present. I wonder if the results would be easier to interpret with a knock-out or knock-down of the protein?

© 2023. Published by The Company of Biologists under the terms of the Creative Commons Attribution License (https://creativecommons.org/licenses/by/4.0/).

We agree that a knock-out allele could be highly informative. Given that CdaH is almost certainly an essential protein, such a knockout would need to be made in the micronucleus and brought to expression by mating of genetic heterokaryons. The germ-line approach is time- consuming and in some loci difficult to execute. The allele that we described has provided a wealth of information by revealing the multiple functions and essential nature of CdaH. Making a CdaH knockout is certainly on our list for future investigations.

The authors used kinase deficient mutants to perform their epistatic experiment. They say "loss of function alleles" p 11. But then, they suggest "that CdaI activity may inhibit CdaH activity that prevent the OP degradation"p12 and later on "CdaH stimulating the CdaI activity…". This is quite confusing. Can the authors perform again these experiments with at least one knock-down or knock-out mutant?

We believe that the two alleles are "loss-of-function" and specifically "loss of kinase activity" based on their recessive character and that in both are substitutions of the highly conserved amino acids in the kinase activity domains. *cdaI-1* affects the substrate binding interface (Jiang et al Genetics, 2017), while *cdaH-1* impacts the highly conserved APE motif that dock the activation loop to the F-helix, a key scaffold element in the kinase domain core (Modi and Dunbrack PNAS USA 2019). However, we do not know whether the two alleles are null and therefore testing an epistasis is problematic. Nonetheless, we constructed double mutants because a potential genetic interaction can be interpreted assuming that both alleles represent at the least a partial loss of function. We indeed found that *cdaI-1* suppresses the OP degradation phenotype conferred by *cdaH-1*, which led us to propose that CdaI activity inhibits CdaH activity in the context of BB resorption. This model is based on an assumption that the mutant CdaH protein has residual activity at least under condition when the inhibition imposed by CdaI is reduced by the *cdaI-1* mutation.

Given the essential role of the two proteins, constructing knockout strains will be time consuming and therefore is a future goal as mentioned above.

3) The CdaH protein seems closer to the human Stk36 protein, but the function seems different.

At this time we would not exclude a possibility that the role of Fused in intracellular patterning revealed in our study is conserved at some level. Intracellular patterns are common if not ubiquitous in animal cells but not as elaborate and amenable to investigation as those in ciliates. It is also possible that Fused kinases share a conserved molecular function (e.g. phosphorylating a kinesin as already established in Drosophila and Arabidopsis) and that this activity is a part of diverse higher level cellular processes including signaling during development and cell polarity-related phenomena. Finally, it is possible that ciliates during their evolution have recruited Fused for lineage specific functions in intracellular patterning and that in ciliates Fused acts by phosphorylating ciliate-specific substrates.

An alignment between the Tetrahymena protein and other Fused protein found during evolution will be necessary for the reader to better interpret the results?

In the revised manuscript we included an alignment of CdaH and a few Fused orthologs as well as a phylogenetic tree that strongly supports our conclusion that CdaH is a Fused kinase (Figure S1). Specifically, we performed a phylogenetic analysis using all known human kinases and CdaH as well as *Tetrahymena* ULK4-related kinases. The analysis strongly supports orthology between CdaH and Fused/Stk36 kinases.

4) The authors mention an other Fused-like protein in the Tetrahymena genome (TTHERM_726480), which appears to be closer to the mammalian ULK4. Could this protein have redundancy with the CdaH one? Similarly, an alignment would be necessary.

In the revised manuscript, we included the two ULK4-releated kinases of Tetrahymena in the phylogenetic analysis (Figure S1). The results establish that CdaH is an ortholog of Fused/Stk36 while TTHERM_00756520 and TTHERM_00726480 are orthologs of ULK4. Regarding the potential

© 2023. Published by The Company of Biologists under the terms of the Creative Commons Attribution License (https://creativecommons.org/licenses/by/4.0/).

redundancy among the above Tetrahymena kinases, if it exists, it is not sufficient to provide for the functionality of CdaH, as the phenotype of its loss of function (*cdaH-1*) is severe (lethal).

5) Regarding the lack of defects in the axonemal structure in the CdaH-1 mutant: It was observed previously in one PCD-affected individual with situs solitus with a loss-of-function mutation in Stk36 gene, that the axonemal structure lacked the central pair. I wonder if the normal axonemal structure is because the electron microscopy was done randomly and not on cells at the onset of cytokinesis (Figure1 stage 3)? Have the basal bodies of the posterior hemi-cell, showing the most significant staining, be analyzed.

It is very unlikely that the *cdaH-1* phenotype is related to ciliary axonemes. We and others have analyzed a number of null mutations in conserved ciliary proteins including IFT components that lead to a complete loss of all ciliary axonemes. Consistently, severe ciliary deficiencies have not been reported to affect cortical pattern except for the cytokinesis defect that occurs at the stage of scission as a result of lack of rotokinesis. Even the most severe ciliary mutants complete cortical subdivision and even cytokinesis when external mechanical force is applied to aid in the scission. Also, defects in the positioning of the oral apparatus have not been reported in ciliary mutants. Not surprisingly we have not encountered 9+0 cilia in the *cdaH-1* mutant at the restrictive temperature. In the legend of figure S3 we added a statement that the TEM images are representative and that we examined a total of 83 cross-sections of mutant axonemes on multiple cells.

While, CdaH localizes to a subset of BBs, this is a common property of all signaling pattern-regulators studied so far, which reflects the roles of BBs beyond their function as nucleators of cilia. We agree with the reviewer that CdaH could be involved with the basal bodies specifically in the subequatorial region, and this idea could be pursued in the future in a more systematic ultrastructural study. However, before embarking on such a major project, we would like first to learn about the potential substrates of CdaH-mediated phosphorylation as their nature could guide us toward specific pathways or structures (e.g. the key substrate could be a BB protein). Our goal for this paper was more limited, given the well described involvement of Fused with cilia, and in particular with the central pair, we wished to determine whether in addition to its role in patterning and cytokinesis CdaH also plays a role in cilia assembly.

Minor comments

1) The temperature vary in the experiments between 38° and 39°. Why? Is it a writing mistake?

The majority of experiments with *cdaH-1* mutants were done at 39°C. One exception are the rescue experiments that were performed at 38°C. At both temperatures the mutant cells arrest in division.

2) Figure 4: it is disturbing to see the the CdaH-GFP signal in red while the centrin dot are in green. Is it some reasons for this choice? Can the authors put the GFP signal in green and the centrin signal in red? Same for Figure 6 G-G,'H-H', Figure 7, S2 and S5.

We changed the color channels to use the colors consistently. In the revised figures centrin is shown in green and CdaH-GFP in magenta.

3) Electron microscope method is absent.

In the Materials and Methods we cited the TEM protocol used by Vasudevan et al 2015.

Reviewer 3 Advance Summary and Potential Significance to Field:

Lee et al. describe the identification of the causal gene for a Tetrahymena patterning mutant cdaH as a Fused/Stk36 kinase. Furthermore, they go on to describe the localization of CdaH throughout the cell division cycle and characterize genetic interactions with other known patterning genes, cdaA and cdaI. The authors implicate cdaH in working to position the division plane, appearing even before actin to the site of division, in a CdaA/Cyclin E-dependent process. CdaH also seems to interact with CdaI to control the formation of membranelles during OA patterning. Overall, I think this is a very interesting paper that adds

© 2023. Published by The Company of Biologists under the terms of the Creative Commons Attribution License (https://creativecommons.org/licenses/by/4.0/).

to the growing number of patterning genes that appear to be from highly conserved pathways throughout Eukaryotes. These findings represent a glimpse into different evolutionary specializations of these genes, or potentially some of their ancestral functions. I recommend this manuscript for publication aker minor revisions.

Reviewer 3 Comments for the Author:
Major Comments:
1. On page 6 the authors discuss mapping the causative gene and rescuing the phenotypes by inserting functional copies of either of the two closely linked genes. They say that TTHERM_00649260 were not observed to rescue the mutant phenotype, but these data don't appear to be shown in Figure 3. All of the rescues shown, and discussed in the figure legend, are rescues of TTHERM_01345780.

In the revised manuscript we clarified that we obtained a total of 2 rescue clones with the TTHERM_01345780 PCR fragment and none with the TTHERM_00649260 PCR fragment. Given the low frequency, in response to the reviewer comment, we designed another "rescue-attempt" experiment using a more statistically-sound approach. We constructed plasmids carrying the 5' UTR of TTHERM_01345780, a linked selectable marker, an MTT1 promoter followed by a portion of the coding region that either spanned the variant site or terminated upstream and thus did not cover the variant site. 100% of transformed *cdaH-1* mutants that integrated the long fragment (containing the wild-type codon at the variant site) were rescued while none of the transformants that integrated the short fragment lacking the sequence around the variant site were rescued (n=96). Thus, there is no doubt regarding the identification of *CDAH* as *TTHERM_00649260*. These new data are included in the revised manuscript.

2. There are a few places the authors should list the N for experiments where representative images are shown. For example, in Figure 5 the authors show kymogram and FRAP data but it is unclear how many cells were imaged to make these conclusions.

In the revised manuscript we stated that the FRAP assay performed on the CdaH-GFP ring documented in Figure 5G-G" is representative of trials on a total 3 cells, while the FRAP assay on the posterior streaks shown in Figure S4C-C' is representative of trials on 5 cells.

3. On page 12, referring to data in Figure 8, the authors are making claim that there is a significant enhancement of the "excessive number of membranelles" phenotype in the cdaH-1;cdaI-1 double mutants as compared to cdaI-1 alone. It seems the authors are referring to an increased penetrance of the phenotype in the double, rather than an enhancement of the phenotype where one might predict more membranellar bands to form. I think the language could be made clearer here, but if there is an increase in the number of membranellar bands seen in their data (which might be the case in the representative images), that would be an interesting thing to quantify.

This is an excellent suggestion and indeed our statement about phenotypic enhancement was not accurate. We now quantified the number of membranelles and found no difference between the single *cdaI-1* and double *cdaI-1+cdaH-1* mutants. Thus, we agree with the reviewer that the difference is in the phenotype penetrance (Fig. 8J). We added the new data (Figure 8K) and toned down our conclusions. We now think that the potential role of CdaH in the "membranelle counting" could be more indirect, e.g. due to its role in the resorption of basal bodies.

4. Also referring to the data in Figure 8J, the authors should perform an appropriate statistical test to demonstrate a significant increase in the % of cells displaying the "Additional membranelle" phenotype in the double-mutant as compared to the cdaI-1 single mutant.

The membranelle count averages (Fig. 8K) were compared using an unpaired t-test (Two-tailed) and were found to be not different at $p<0.05$.

Minor Comments:
1. Authors should avoid the use of Red and Green colors together, or entirely, which would increase accessibility to colorblind readers. Good options are described in an ASCB article (https://www.ascb.org/science-news/how-to-make- scientific-figures-accessible-to-readers-

© 2023. Published by The Company of Biologists under the terms of the Creative Commons Attribution License (https://creativecommons.org/licenses/by/4.0/).

with-color-blindness/)

We replaced the red color with magenta in all figures.

2. Color choices in the figures could be more consistent. For example, sometimes actin is shown red, other times it is green. In Figure 6 Actin is shown as red in panels A-E” but is green in panels G-H”. While the figure is clearly labeled, consistent colors would help to avoid reader confusion.

We have now revised the color channels to use them consistently across all figure panels.

3. Movie 1 and Movie 2 should have time-stamps and scale bars

As suggested, we added time-stamps and scale bars to movies.

4. On page 4, there is a typo where “sculpturing” should probably be “sculpting”

We made the change as suggested.

5. Figure 6 Legend: Typo, “labeled with polyclonal anti-Act1 actin antibodies (red0…” where the 0 should be a).

We corrected the typo.

Second decision letter

MS ID#: JOCES/2023/261256

MS TITLE: Global and local functions of a Fused/Stk36 kinase, CdaH, in intracellular patterning in Tetrahymena

AUTHORS: Chinkyu Lee, Wolfgang Maier, Yu-Yang Jiang, Kentaro Nakano, Karl F Lechtreck, and Jacek Gaertig
ARTICLE TYPE: Research Article

It is my pleasure to tell you that your manuscript has been accepted for publication in Journal of Cell Science, pending standard ethics checks. Referee reports are appended below. It was a delight to handle your manuscript and all reviewers concur on the importance and quality of your work.

Reviewer 1

Advance summary and potential significance to field

Dear Sirs The authors have answered to all my questions and changed the manuscript according to my previous major concerns.
Also, they coherent answered to other referees.
In conclusion the manuscript is now suitable for publication.

Comments for the author

Dear Sirs The authors have answered to all my questions and changed the manuscript according to my previous major concerns.
Also, they coherent answered to the other referees.
In conclusion the manuscript is now suitable for publication.

© 2023. Published by The Company of Biologists under the terms of the Creative Commons Attribution License (https://creativecommons.org/licenses/by/4.0/).

<u>Reviewer 2</u>

Advance summary and potential significance to field

In this manuscript entitled “Global and local functions of a Fused/Stk36 kinase, CdaH, in intracellular patterning in Tetrahymena”, Lee and colleagues analyses a thermosensitive mutant CdaH-1 in Tetrahymena. This paper is the third paper of a series of Tetrahymena mutant analysis (Jiang et al, 2017 and Jiang et al, 2020). As previously, the paper characterized the phenotype of the mutant at the restrictive temperature, identified the mutated gene with its mutated amino-acid, and localized the protein as a GFP-fusion protein. Because the phenotypes of this mutant show similarities to the two previously identified mutants (Jiang et al, 2017 and Jiang et al, 2020), the authors investigates epistatic relationships between the three mutants.

To conclude, the paper is interesting and highlight the function of a conserved kinase in the Tetrahymena patterning.

Comments for the author

The authors have mostly answer my questions. Only the knock-out has not been done since this method is time consuming. I understand that those knock-out were out of the scope of the paper. I recommend publication of this paper.

<u>Reviewer 3</u>

Advance summary and potential significance to field

The authors describe the identification of the causal gene for a Tetrahymena patterning mutant cdaH as a Fused/Stk36 kinase. Furthermore, they go on to describe the localization of CdaH throughout the cell division cycle and characterize genetic interactions with other known patterning genes, cdaA and cdaI. The authors implicate cdaH in working to position the division plane appearing even before actin to the site of division, in a CdaA/Cyclin E-dependent process. CdaH also seems to interact with CdaI to control the formation of membranelles during OA patterning. Overall, I think this is a very interesting paper that adds to the growing number of patterning genes that appear to be from highly conserved protein families throughout Eukaryotes. These findings represent a glimpse into different evolutionary specializations of these genes, or potentially some of their ancestral functions. Given the authors' ability to thoroughly address mine and other reviewer comments I would recommend this manuscript for publication at this time.

Comments for the author

I have no additional major or minor comments. The authors have done a nice job addressing the vast majority of major/minor comments and I have not identified any new or lingering issues.
s

© 2023. Published by The Company of Biologists under the terms of the Creative Commons Attribution License (https://creativecommons.org/licenses/by/4.0/).
